# Anti-dissolution Pt single site with Pt(OH)(O_3_)/Co(P) coordination for efficient alkaline water splitting electrolyzer

**DOI:** 10.1038/s41467-022-31406-0

**Published:** 2022-07-02

**Authors:** Lingyou Zeng, Zhonglong Zhao, Fan Lv, Zhonghong Xia, Shi-Yu Lu, Jiong Li, Kaian Sun, Kai Wang, Yingjun Sun, Qizheng Huang, Yan Chen, Qinghua Zhang, Lin Gu, Gang Lu, Shaojun Guo

**Affiliations:** 1 School of Materials Science and Engineering, Peking University, Beijing, China; 2 School of Physical Science and Technology, Inner Mongolia University, Hohhot, China; 3 Shanghai Synchrotron Radiation Facilities, Shanghai Institute of Applied Physics, Chinese Academy of Science, Shanghai, China; 4 Department of Chemistry, Tsinghua University, Beijing, China; 5 Beijing National Laboratory for Condensed Matter Physics, Institute of Physics, Chinese Academy of Sciences, Beijing, China; 6 Department of Physics and Astronomy, California State University Northridge, Northridge, CA USA

**Keywords:** Electrocatalysis, Electrocatalysis, Nanoscale materials

## Abstract

As the most well-known electrocatalyst for cathodic hydrogen evolution in water splitting electrolyzers, platinum is unfortunately inefficient for anodic oxygen evolution due to its over-binding with oxygen species and excessive dissolution in oxidative environment. Herein we show that single Pt atoms dispersed in cobalt hydrogen phosphate with an unique Pt(OH)(O_3_)/Co(P) coordination can achieve remarkable catalytic activity and stability for oxygen evolution. The catalyst yields a high turnover frequency (35.1 ± 5.2 s^−1^) and mass activity (69.5 ± 10.3 A mg^−1^) at an overpotential of 300 mV and excellent stability. Mechanistic studies elucidate that the superior catalytic performance of isolated Pt atoms herein stems from optimal binding energies of oxygen intermediate and also their strong electronic coupling with neighboring Co atoms that suppresses the formation of soluble Pt^*x*>4^ species. Alkaline water electrolyzers assembled with an ultralow Pt loading realizes an industrial-level current density of 1 A cm^−2^ at 1.8 volts with a high durability.

## Abstract (web summary)

Platinum is the most active catalyst for cathodic hydrogen evolution, however it is inefficient for anodic oxygen evolution. Here, authors examine Pt single sites in cobalt hydrogen phosphate as oxygen evolution catalysts in water splitting electrolyzers.

## Introduction

Development of efficient and durable electrocatalysts for both cathodic hydrogen evolution reaction (HER) and anodic oxygen evolution reaction (OER) is essential to fulfill the promises of water splitting electrolyzers^1,2^. More challenging between the two, OER suffers from particularly sluggish kinetics, thus demanding high-performance catalysts to lower the reaction overpotential for practical applications^3,4^. Over the past decades, noble metals, such as Ir^5–7^ and Ru^8,9^ have been extensively explored as potential OER catalysts, but their turnover frequency (TOF) and mass activity (MA) values remain less than satisfactory owing to their insufficient atom-utilization efficiencies, as exemplified in IrO_2_ (0.01 s^−1^)^10^, IrO_2_/GCN (0.17 s^−1^)^11^, Cr_0.6_Ru_0.4_O_2_ (0.229 A mg_Ru_^−1^)^12^ and Ru_1_-Pt_3_Cu (0.779 A mg_Ru_^−1^)^13^. Moreover, although significant progress has been made in rotating disk electrodes, much less is known of an integral device configuration^14^.

Platinum (Pt) is the best known elemental HER catalyst at the cathode of water splitting electrolyzers thanks to its optimal hydrogen absorption energy^15,16^. However, Pt is less efficient for OER at the anode due to its overly strong bonding with oxygen intermediates^1,17^. In addition, Pt is readily oxidized to soluble high-valence Pt^*x*>4^ derivatives (*e.g*., PtO_3_) in an excessively oxidative environment at the anode potential of >1.4 V versus a reversible hydrogen electrode (RHE)^18–20^. This in turn could trigger dissolution of active Pt species, resulting in a sharp decrease in catalytic activity^18,20,21^. Therefore, the rational design of a highly efficient and dissolution-resistant Pt-based OER catalyst requires fine control over the local coordination environment and electronic structure of Pt, lowering the OER overpotential and simultaneously suppressing the over-oxidation of active Pt species during electrolysis. To go further, considering the noble nature of Pt, downsizing the nanometre-scale to single-atoms could effectively decrease Pt usage and maximize atomic utilization efficiency. However, despite tremendous efforts have been dedicated to tuning the electronic structure and coordination of Pt in recent decades^15–17^, whether Pt sites can be tailored to exhibit true state-of-the-art Ir/Ru-like activity on a TOF or MA basis and to what degree they are stable in the OER still remains an open question.

Herein, we present a class of highly active and stable OER catalysts with atomically dispersed Pt sites embedded in cobalt hydrogen phosphate (CoHPO) support with Pt(OH)(O_3_)/Co(P) coordination. Over a long period of time, platinum has been neglected as a catalyst for the OER as its nanoparticle was tested to be almost inert^1,21^. In this work, surprisingly, the Pt-based single-site catalysts display 2–4 orders of magnitude higher TOF and MA than Pt/C and Pt nanoparticles on CoHPO and the state-of-the-art Ir/C electrocatalysts, and also show excellent stability. Combining a series of experimental studies and density functional theory (DFT) calculations indicate that the special coordination structure of monatomic Pt sites coupled with strong electronic coupling between isolated Pt atoms and surrounding Co atoms are the origin of much enhanced electrocatalytic activity and stability. Impressively, the catalysts also show remarkable HER performance with a TOF as high as 12.8 s^−1^ *per* Pt at −100 mV, about 41 times greater than that of commercial Pt/C. We further assemble an anion-exchange-membrane water electrolyser (AEMWE) using this bifunctional catalyst as both anode and cathode with an ultralow total mass loading of 0.029 mg_Pt_ cm^−2^. The AEMWE achieves an industrial current density of 1 A cm^−2^ at a low cell voltage of 1.8 volts without *iR*-correction and durability of more than 100 h, which delivers two-order of magnitude higher performance than commercial Pt/C and Ir/C in terms of mass density and precious metal utilization efficiency.

## Results

### Synthesis and structural characterizations

**Fig. 1:**
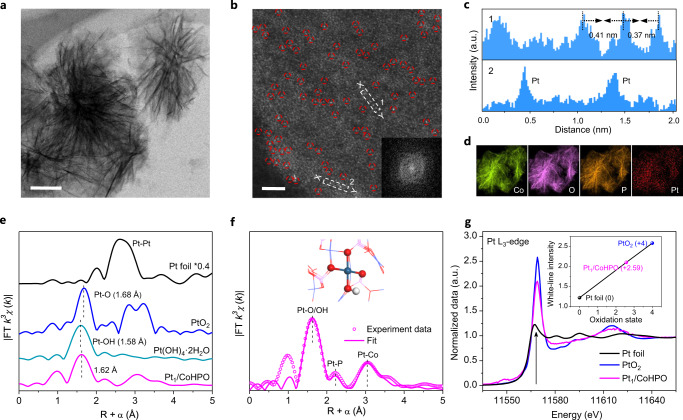
Morphology and fine-structure characterizations of the Pt_1_/CoHPO catalyst. **a** TEM image. Sale bar, 0.5 μm. **b** Representative magnified HAADF-STEM image, showing that only Pt single atoms are present in the CoHPO support (marked by red dotted circles). *Inset*, the FFT image. Sale bar, 2 nm. **c** Atomic line-scanning intensity profiles along the X-Y marked by 1 and 2 in **b**. **d** Elemental mapping. **e** *k*^3^-weighted Fourier-transformed (FT) of EXAFS spectra at *R* space. **f** Fitting curve of EXAFS spectra at *R* space. *Inset*, showing the optimized atomic model of Pt_1_/CoHPO, Pt (dark cyan), Co (blue), O (red), P (pink) and H (white). **g** XANES spectra at the Pt *L*_3_-edge. *Inset*, the fitted average valence state of Pt from XANES spectra.

Atomic platinum was anchored on CoHPO support (Supplementary Figs. 1 and 2) by mean of an icing-assisted photochemical reduction. In this process, PtCl_6_^2−^ precursor was effectively inhibited to migrate, avoiding the nucleation and growth of platinum species into clusters or particles. Inductively coupled plasma (ICP) spectroscopy result verifies that the platinum concentration on Pt_1_/CoHPO is 0.57 wt.%. Due to low incorporation of platinum, the X-ray diffraction (XRD) pattern exhibits no perceptible change compared to original CoHPO (Supplementary Fig. 2b). Nanosheet-assembled flower-like Pt_1_/CoHPO is observed from the transmission electron microscopy (TEM) image (Fig. 1a), without any discernible metallic nanoparticles. Aberration-corrected high-angle annular dark-field (HAADF) scanning TEM (STEM) image (Fig. 1b and Supplementary Fig. 3) show platinum atoms (marked by dashed red cycles) are atomically dispersed on amorphous CoHPO (inset of Fig. 1b). Moreover, the atomic intensity profiles along with the direction of X-Y (Fig. 1c) uncover that each platinum atoms are separated by at least 0.37 nm, exceeding the platinum-effective atomic radius, further corroborating the single-site platinum on the supports. The composition analysis by STEM elemental mapping at larger scale reveals the homogenous dispersion of platinum species in Pt_1_/CoHPO (Fig. 1d).

X-ray absorption fine structure spectroscopy (XAFS) at Pt L_3_-edge was used to investigate the local coordination environment of single-atom Pt_1_ in Pt_1_/CoHPO. The Fourier transform (FT) of extended XAFS (EXAFS) oscillations (Fig. 1e) reveal that Pt_1_/CoHPO exhibits a major peak at ~1.62 Å, shorter than the Pt-O peak at ~1.68 Å for the PtO_6_ octahedra in PtO_2_, and slightly larger than the Pt-OH peak at ~1.58 Å for the Pt-OH_6_ octahedra in Pt(OH)_4_, which can thus be assigned to a mixture of Pt-O and Pt-OH bonding. The coexistence of O^2−^ and OH^−^ species was also revealed by O 1 s X-ray photoelectron spectroscopy (XPS) spectrum (Supplementary Fig. 4). In addition, Pt_1_/CoHPO reveals two minor signals at ~2.22 and ~3.05 Å, attributed to Pt-P and Pt-Co bonding, respectively (Supplementary Fig. 5). Compared with Pt foil and PtO_2_, there are no characteristic peaks for Pt-Pt or Pt-O-Pt (clustered platinum oxides) scatterings in Pt_1_/CoHPO, consistent with the result of HAADF-STEM images. Based on the EXAFS best-fitting analyses (Supplementary Table 1), the Pt_1_ atomic environment within Pt_1_/CoHPO consists of three O atoms and one OH atom in first coordination shell, and four Co and P atoms in second coordination shell (inset of Fig. 1f). We further identified atomic-site structures of Pt_1_/CoHPO by X-ray absorption near-edge structure (XANES) simulations, which has high sensitivity to the three-dimensional arrangement of atoms^22^. The XANES profiles of Pt_1_/CoHPO can be reproduced fairly well by the structure of a Pt(OH)(O_3_)/Co(P) moiety embedded in a cobalt hydrogen phosphate support (Supplementary Fig. 9). The interatomic distances predicted by density Functional theory (DFT) are also fully consistent with the structural parameters obtained by FT-EXAFS fitting (Supplementary Fig. 8 and Table 1).

The XANES and XPS measurements were further performed to study the chemical state of Pt_1_ in Pt_1_/CoHPO and the electronic coupling between Pt_1_ and substrate. The normalized XANES spectra show that the white line intensity (black arrow) of Pt_1_/CoHPO is between platinum powder (0) and PtO_2_ (+4) (Fig. 1g). The fitted valence state of platinum from XANES spectra is +2.59 (inset of Fig. 1g), being in agreement with the Pt 4 *f* XPS analysis (Supplementary Fig. 10). Noticeably, a negative shift (0.5 eV) in the Co 2*p* XPS spectra (Supplementary Fig. 11) is observed upon Pt loading. The increase of Co valence state in Pt_1_/CoHPO can be attributed to the noble Pt with higher electronegativity, which is attracting electrons from Co through the Co-O-Pt bonds, being in accord with the fact that Pt possess a valance state lower than its initial salt K_2_PtCl_6_, implying the strong electronic interactions of Pt and Co atoms^23^.

As a control, other various single-atom metals anchored on CoHPO (M_1_/CoHPO), such as Ir_1_, Ru_1_, Ni_1_ and Fe_1_, were also synthesized using similar procedure with that of Pt_1_/CoHPO (Methods). The atomically dispersed nature of M_1_/CoHPO was demonstrated by high-resolution STEM and XAFS spectra (Supplementary Figs. 12–15). Meanwhile, as a comparison, the CoHPO with Pt nanoparticles of ~3 nm (1.15 wt.%) was also synthesized via a NaBH_4_-reduction method (denoted as Pt_NP_/CoHPO, Supplementary Fig. 16).

### Electrochemical OER

**Fig. 2:**
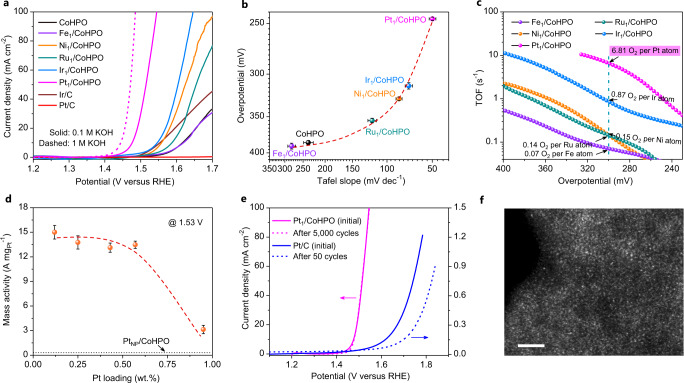
Electrochemical OER performance. **a** Polarization curves of CoHPO and different M_1_/CoHPO catalysts tested in 0.1 M and 1 M KOH solutions. Reference samples of Ir/C and Pt/C are also included for comparison. **b** OER data analysis of overpotentials (obtained from the polarization curves at 10 mA cm^−2^ current density) and Tafel slopes in 0.1 M KOH. **c** TOFs curves of different M_1_/CoHPO catalysts based on the loading amounts of metals at different overpotentials. **d** Mass activities of Pt_1_/CoHPO with different Pt loadings at 1.53 V *vs*. RHE. **e** The polarization curves of Pt_1_/CoHPO and Pt/C before and after 5000 (for Pt_1_/CoHPO) and 50 (for Pt/C) potential cycles tested in 0.1 M KOH. **f** A high-resolution HAADF-STEM image of Pt_1_/CoHPO after the durability test, in which the bright contrast spots present the Pt single atoms. Scale bar, 2 nm. Error bars in **b**, **d** represent the average values (mean ± s.d., *n* = 3).

The OER polarization curves in O_2_-saturated 0.1 M KOH (Fig. 2a) reveal that the Pt_1_/CoHPO exhibits an unexpected catalytic activity with a low overpotential of 246 mV (209 mV in 1 M KOH) at 10 mA cm^−2^, much lower than those of state-of-the-art Ir/C (344 mV) and other M_1_/CoHPO that include Ir_1_ (313 mV), Ru_1_ (355 mV), Ni_1_ (328 mV) and Fe_1_ (390 mV), whereas the nanoparticle counterparts Pt/C and Pt_NP_/CoHPO (Supplementary Fig. 18) show very low catalytic activities. The overpotential *vs*. Tafel slope (Fig. 2b and Supplementary Fig. 19) and electrochemical impedance (Supplementary Fig. 20) further reveal the more favorable OER kinetics of Pt_1_/CoHPO, in which the Pt_1_/CoHPO shows the lowest Tafel slope (49.8 mV dec^−1^) and charge-transfer resistance (8.1 Ω). We note that the OER activity of CoHPO support is two orders of magnitude lower than that of Pt_1_/CoHPO (Supplementary Fig. 18), suggesting that the atomically dispersed Pt_1_ species plays a crucial role in the observed high OER activity. Moreover, Pt_1_/CoHPO exhibits a TOF value of 6.81 ± 0.13 s^−1^ per Pt atoms and MA of 13.5 ± 0.3 A mg^−1^_Pt_ at 300 mV in 0.1 M KOH, four orders of magnitude larger than that of Pt/C (7.6 × 10^−4^ s^−1^, 1.5 × 10^−3^ A mg^−1^_Pt_) and 52-times larger than that of Pt_NP_/CoHPO (0.13 s^−1^, 0.26 A mg^−1^_Pt_, Supplementary Fig. 18b), and also superior to those of other M_1_/CoHPO (Fig. 2c) and state-of-the-art Ir/C (0.027 s^−1^, 0.056 A mg^−1^_Ir_). When evaluated in 1 M KOH as the electrolyte, the TOF and MA values increase to as high as 35.1 ± 5.2 s^−1^ and 69.5 ± 10.3 A mg^−1^_Pt_ at 300 mV (Fig. 2a and Supplementary Fig. 18c, d), respectively, which are superior to most of the state-of-the-art single-atom oxygen-evolving catalysts and Ir, Ru-based catalysts reported to date (Supplementary Table 3).

The atomic dispersion of platinum is vital to the high activity of Pt_1_/CoHPO in the OER. Figure 2d presents the MAs at 1.53 V as a function of the Pt loadings. For the catalysts with low Pt loadings, there are subtle differences in MAs until the loading reaches to ~0.95 wt.%, namely Pt_1_Pt_NP_/CoHPO, with mixed Pt single atoms and nanoparticles (Supplementary Fig. 21). Specifically, MAs of single-atom samples with a low Pt loadings (0.12, 0.25, 0.43 and 0.57 wt.%) all exceed 13 A mg_Pt_^−1^ at 1.53 V, decreases to 3.1 A mg_Pt_^−1^ for Pt_1_Pt_NP_/CoHPO, and further sharply decreases to 0.26 A mg_Pt_^−1^ for Pt_NP_/CoHPO (Supplementary Fig. 16). These results clearly show that the single atomic Pt_1_ is responsible for the high OER performance of Pt_1_/CoHPO.

We further found that Pt_1_/CoHPO was quite stable in long-term catalysis. The OER polarization curves obtained before and after the accelerated durability tests of 5000 potential cycles almost overlap, well surpassing that of Pt/C (Fig. 2e). The stability was further demonstrated by the negligible increase in potential during the 48 h test (Supplementary Fig. 22). Moreover, unlike the ~60% dissolution of Pt/C catalysts, no Pt species were detected in Pt_1_/CoHPO by ICP in electrolyte after the chronoamperometry test (Supplementary Table 4), suggesting the high structural stability of single atomic Pt_1_ in the CoHPO with strong resistance to dissolution under harsh OER conditions. Atomic-resolution HAADF-STEM images and elemental mappings (Fig. 2f and Supplementary Fig. 23) further confirm the uniformly distributed single atomic Pt_1_ on the CoHPO after stability test, without noticeable agglomeration of larger Pt species.

### Insights into the underlying OER mechanism

**Fig. 3:**
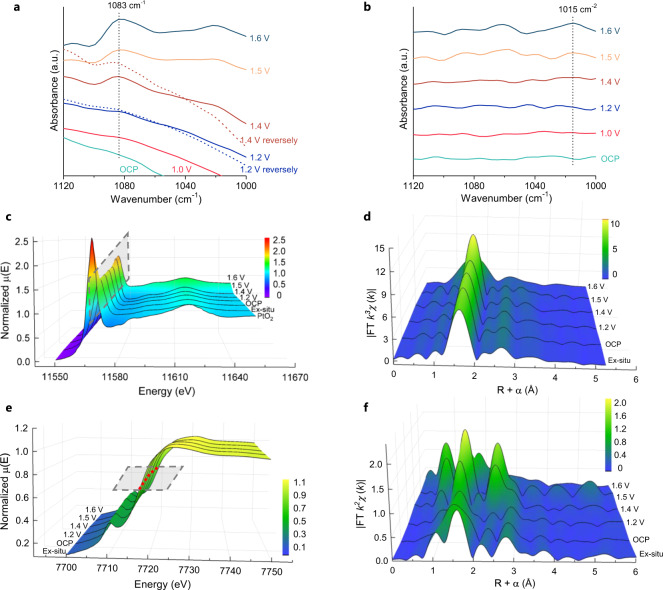
*Operando* ATR-FTIR and XAFS spectra of catalysts under different applied potentials. **a**, **b** *Operando* potential-dependent ATR-FTIR spectra for Pt_1_/CoHPO (**a**) and CoHPO (**b**). **c**–**f**, *Operando* potential-dependent XAFS spectra of the XANES (**c**, **e**) and FT-EXAFS (**d**, **f**) at the Pt L_3_-edge (**c**, **d**) and Co K-edge (**e**, **f**) of Pt_1_/CoHPO.

The potential-dependent *operando* attenuated-total-reflection (ATR) Fourier-transform infrared (FTIR) spectroscopy was performed to probe the mechanistic pathway and possible role of single-atom Pt_1_ in Pt_1_/CoHPO. No absorption bands were detected on either Pt_1_/CoHPO or CoHPO at open circuit potential (OCP). For Pt_1_/CoHPO, no obvious band was observed at potentials of 1.0 and 1.2 V versus RHE, which are more negative than OER thermodynamic potential. A prominent absorption vibration band at ~1083 cm^−1^, assigned to the stretching vibration of superoxide species (-O-O-) on the Pt surface (refs. ^24,25^), appears at a potential of 1.4 V and gradually strengthens in intensity with the augment of the applied potentials (from 1.4 V to 1.6 V). When 1.2 and 1.4 V were reversely applied to Pt_1_/CoHPO, the characteristic bands intensity was identical with the original ones (Fig. 3a). These results demonstrate the formation of a surface intermediate superoxide OOH_ads_ species on the Pt_1_ sites during the OER^26^, affirming that single-atom Pt_1_ species in Pt_1_/CoHPO act as the dominating active site for the OER. Meanwhile, the emergence of OOH_ads_ intermediate suggests that an adsorbate evolution mechanism (AEM) pathway rather than a lattice oxygen activation mechanism (LOM) pathway dominated O_2_ generation over the Pt_1_/CoHPO, which improved its structural stability under harsh OER conditions (Supplementary Fig. 24, refs. ^27,28^). By contrast, for CoHPO, only a very weak band at ~1015 cm^−1^ was observed when potential was applied (Fig. 3b), which corresponds to the vibrational band of Co-OOH_ads_ (ref. ^29^), suggesting insufficient OOH_ads_ generation and sluggish kinetics on the cobalt sites.

We further applied *operando* XAFS techniques to investigate the potential-dependent oxidation of Pt_1_ during OER (Supplementary Fig. 25). With the potential increased from OCP to 1.6 V, the oxidation state of Pt_1_ in Pt_1_/CoHPO appeared relatively stable, lower than that of reference PtO_2_ (+4), as revealed by the essentially identical normalized white-line intensities (Fig. 3c) and local coordination (Fig. 3d) at the Pt L_3_-edge. In contrast, an appreciably shift to higher energy in the absorption threshold of the Co K-edge (Fig. 3e) indicates a decrease of *d*-band occupy states for Co atoms in the CoHPO substrate, probably attributed to the electron transferring from adjacent Co to Pt_1_. This is further corroborated by surface XPS analysis (Supplementary Fig. 25), in which Pt_1_ almost kept the initial oxidation state after running a chronoamperometry test, without transforming into an unstable phase of Pt^*x*>4^ derivative, considering the possible charge compensation from Co to Pt_1_ induced by the strong metal-support interactions, thereby preventing the over-oxidation and dissolution of Pt_1_. In addition, a shrinkage of the Co-O bond is also observed with increasing the potential (Fig. 3f), which could further fix the isolated Pt_1_ atoms on the surface of Pt_1_/CoHPO, avoiding possible migration and agglomeration during OER.

**Fig. 4:**
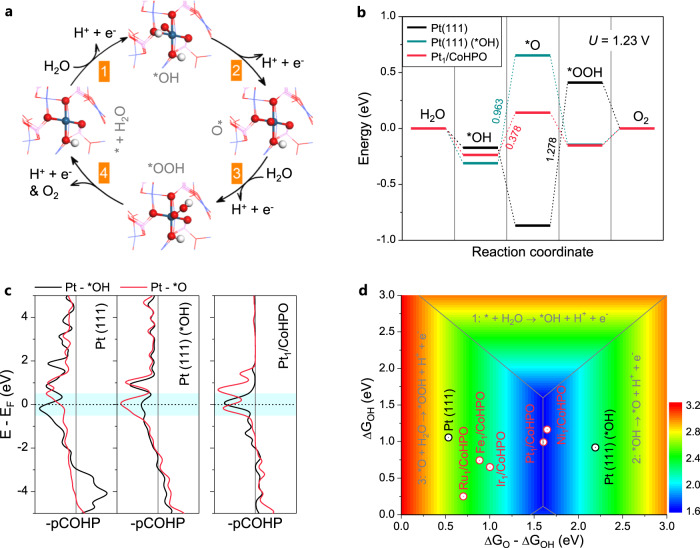
DFT simulations of catalytic activity and electronic structure. **a** The OER reaction pathway on Pt_1_/CoHPO. The adsorption structures of intermediates on the HO_1_-Pt_1_-O_3_/Co(P) sites are shown in the *insets*. Cyan, red, and white spheres represent Pt, O, and H atoms, respectively. **b** The OER free energy diagram for Pt (111), OH-covered Pt (111), and Pt_1_/CoHPO surfaces at equilibrium potential of 1.23 V. **c** The projected crystal orbital Hamilton population (pCOHP) for Pt-O bond in the *OH and *O adsorbed Pt (111), Pt (111) (*OH), and Pt_1_/CoHPO surfaces. Bonding and antibonding states are shown on the right and left, respectively. **d** Overpotential contour map in terms of the free energies of *OH and difference between *O and *OH.

Theoretical investigations based on first-principles calculations were further implemented to rationalize the observed activity and dissolution resistance of single-atom Pt sites in Pt_1_/CoHPO. A four-electron AEM reaction pathway proposed in the *operando* ATR-FTIR spectroscopy is considered (Fg. 4a). The OH-covered Pt (111) surface is chosen to model the OER reaction at Pt (111) in an alkaline condition. On the clean Pt(111) surface, we find that the OER overpotential is determined by the adsorption of *OOH intermediate on the surface (Fig. 4b). The over-binding of *O intermediate on the surface yields a high overpotential U_OP_ = 1.278 V. In a sharp contrast, *O binding on the OH-covered Pt(111) surface is too weak due to *O-OH interaction on the surface, which also leads to a high overpotential (U_OP_ = 0.963 V). These results are consistent with the classic volcano picture — binding too strongly or too weakly lower the catalytic activity, which explains why the conventional Pt electrodes are inefficient for OER. On the other hand, thanks to the single-atom Pt sites with Pt(OH)(O_3_)/Co(P) coordination, *O binding on the Pt_1_/CoHPO surface is neither too strong or too weak, resulting in a much lower overpotential U_OP_ = 0.378 V. To paint a complete physical picture, we also calculate the projected crystal orbital Hamilton population (pCOHP) for Pt-O (red) and Pt-OH (black) bonds on the three surfaces^30,31^. Since more bonding states and fewer anti-bonding states yield stronger bonding and vice versa, the pCOHP analysis is directly connected to the binding energy results (Fig. 4c). On clean Pt(111) surface, since pCOHP values near E_F_ for the antibonding states of Pt-O bonds are very low, one would expect strong (or over)-binding of *O. In contrast, on OH-covered Pt(111) surface, a large number of antibonding states is present for the Pt-O bonds, leading to weak binding of *O. Interestingly, on Pt_1_/CoHPO surface, the pCOHP values for the antibonding states of Pt-O and Pt-OH bonds are comparable, suggesting similar binding energies of *O and *OH intermediates, which in turn gives rise to a lower overpotential. Therefore, the binding energy results are consistent with the premises of the *d*-band theory although the latter is not explicitly considered here.

Using the linear scaling relation ΔG_*OOH_ = 0.97ΔG_*OH_ + 3.24 fitted on the basis of the established M_1_/CoHPO models (Supplementary Fig. 27)^32^, we can further construct a volcano plot for OER (Fig. 4d). The constructed volcano is found to provide a reasonable estimate of OER activity on a number of similar catalysts. In particular, Pt_1_/CoHPO sits very close to the top (blue area) of the volcano. On the other hand, Pt(111), Ru_1_, Fe_1_ and Ir_1_/CoHPO surfaces constitute the left-leg (or over-binding) of the volcano, whereas Ni_1_/CoHPO is on the right-leg of the volcano (weak-binding). Pt_1_/CoHPO is predicted to be at the volcano top, agrees well with the experimental observations.

Finally, by Bader charge analysis, we find that the adsorption of an oxygen atom gains ~0.45 e from the Pt_1_/CoHPO surface, with ~0.32 e from the single-atom Pt site, and ~0.13 e from the CoHPO substrate. In other words, the substrate acts as an electron reservoir and donates electrons to the reaction intermediates, which can help suppress the over-oxidation and dissolution of Pt_1_ on the surface, giving rise to much improved stability of Pt_1_/CoHPO catalyst as observed in our experiments.

### Performance in water electrolyzers device

**Fig. 5:**
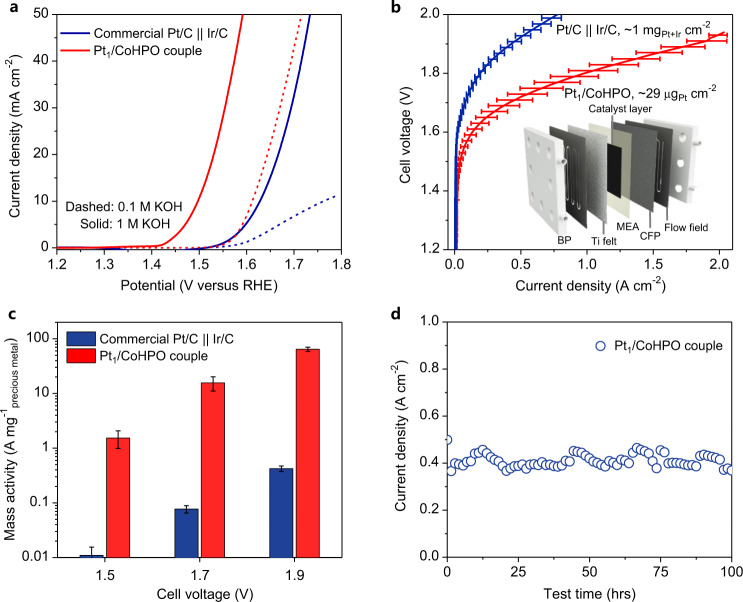
AEMWE performance. **a** Electrocatalytic overall water-splitting properties in a H-type cell of the Pt_1_/CoHPO as both anodic and cathodic catalysts. The benchmark Pt/C + Ir/C as a catalyst is also included for comparison. **b** Electrocatalytic water-splitting properties of the Pt_1_/CoHPO and the benchmark Pt/C + Ir/C measured in an alkaline AEMWE setup operating at 80 °C. *Inset*, a typical single AEMWE setup comprising a membrane electrode assembly (MEA) and bipolar plates (BP) with a flow field are presented, wherein the MEA comprises a gas diffusion layers with a Ti felt and a carbon fiber paper (CFP) at the anodic and cathodic sides, respectively, anodic and cathodic catalyst layers and an anion exchange membrane (AEM). **c** Mass activities comparisons of the Pt_1_/CoHPO and the commercial Pt/C + Ir/C at various cell voltages, showing over two orders of magnitude higher of mass activity for the Pt_1_/CoHPO compared with that of commercial Pt/C + Ir/C. **d** Stability tests of the Pt_1_/CoHPO-based MEA. Error bars in **b**, **c** represent the average values (mean ± s.d., *n* = 3).

In addition to its promising OER property, Pt_1_/CoHPO catalyst also achieves an exceptional low overpotential of 49 mV (in 0.1 M KOH) to deliver 10 mA cm^−2^, a TOF as high as 12.8 s^−1^ at −100 mV as well as superior durability for the HER (Supplementary Figs. 28 and 29). Our Pt_1_/CoHPO is no doubt one of the most efficient bifunctional catalysts toward both OER and HER, exceeding most of the reported state-of-art catalysts (Supplementary Tables 3 and 5). In a proof-of-principle demonstration of its application, we leveraged the bifunctional catalytic activity of Pt_1_/CoHPO, and used it as both anode and cathode for water electrolysis. We first assessed the catalytic properties in a two-electrode lab configuration. Compared to the benchmark Pt/C + Ir/C catalyst, it exhibits a 130 mV smaller potential to achieve 10 mA cm^−2^ as well as enhanced durability (Fig. 5a, Supplementary Fig. 30). The Faradaic efficiency of the production of hydrogen was nearly 100% (Supplementary Fig. 31).

We further integrated our Pt_1_/CoHPO into a membrane-electrode-assembly (MEA) to assemble an alkaline AEMWE device (Supplementary Fig. 32 and 33). Figure 5b shows the water-splitting performance of the Pt_1_/CoHPO-based and the benchmark Pt/C + Ir/C-based AEMWE setup operating in 0.1 M KOH at 80 °C (The error bars show the standard deviation for three independently integrated and tested cells). In addition to an industrial current density of 1 A cm^−2^ at a low cell voltage of 1.8 V (Fig. 5b and Supplementary Fig. 34), the Pt_1_/CoHPO-based MEA containing an ultralow total Pt loading of ~29 μg cm^−2^ (11.4 μg cm^−2^ for cathode and 17.1 μg cm^−2^ for anode) delivers a much higher current density than the MEA that uses benchmark Pt/C + Ir/C (0.2 A cm^−2^ at 1.8 V) in spite of its high precious metal loading of ~1 mg cm_Pt+Ir_^−2^. Mass activities using Pt_1_/CoHPO-based MEA reach 15.7 ± 4.5 and 64.5 ± 5.8 A mg_Pt_^−1^ at 1.7 and 1.9 V (Fig. 5c), respectively, which are more than two-order of magnitude higher than those of commercial Pt/C + Ir/C. Furthermore, the Pt_1_/CoHPO-based MEA can be stably operated for at least 100 h without apparent loss of the current density (Fig. 5d).

## Discussion

In summary, we report a type of anti-dissolution Pt single site with Pt(OH)(O_3_)/Co(P) coordination embedded in cobalt hydrogen phosphate as a highly efficient bifunctional catalyst for both OER and HER catalysis. Studies of the mechanism by employing advanced *operando* ATR-FTIR and X-ray absorption spectroscopic techniques demonstrate that Pt single atoms provide predominant OER active sites and the oxidation state of Pt is kept under 4+ within the OER catalysis potential range. The DFT calculations reveal that the highly enhanced activity and stability on Pt_1_/CoHPO originate from the unique coordination of Pt(OH)(O_3_)/Co(P) sites, and from the strong electronic coupling between isolated Pt atoms and surrounding Co atoms. The resultant charge redistribution not only optimizes the binding energies of oxygenated intermediates, lowers the OER energy barriers, but also prevents the formation of soluble high valence Pt^*x*>4^ by tuning the redox behaviour of Pt. The Pt-based single-atom catalyst with an ultralow total Pt loading enables stable and high-performance water splitting using an alkaline AEM electrolyser as both anode and cathode. This work provides fundamental and technological insights for developing highly efficient catalysts with ultralow Pt loading for renewable energy conversion devices.

## Methods

### Preparation of M_1_/CoHPO

As-prepared CoHPO (20 mg, Supplementary methods) was first ultrasonically dispersed in deionized water (15 mL) in a beaker. A controlled amount of 2 mg mL^−1^ K_2_PtCl_6_ was then dropwise added into the solution, and stirred for 12 h. Subsequently, the solution was rapidly frozen by a liquid nitrogen (−196 °C) bath. The mixed solution could be frozen quickly to form ice, which ensures the homogeneous distribution of Pt precursor in the solution. The frozen ice was then irradiated in a 300 W Xe light (PLS-SXE300D) equipped with 420 nm and IR light filters. After irradiation for about 7 min, the ice layers were naturally melted into solution. The product was collected by centrifugation and washed via ethanol for three times, and dried in an oven at 60 °C overnight. The final Pt loading of Pt_1_/CoHPO was determined by ICP-AES.

The preparation of other metals (Ni, Fe, Ru and Ir) single atoms on the CoHPO support was similar to that of Pt_1_/CoHPO, except that NiCl_2_·6H_2_O, FeCl_2_·4H_2_O, RuCl_3_·*x*H_2_O, and IrCl_3_·*x*H_2_O solutions were used as Ni, Fe, Ru and Ir precursors, respectively.

### Electrochemical measurements

Electrochemical tests were applied using a typical three-electrode cell configuration on CHI 660E instruments. A graphite carbon rod was used as the counter electrode, and a Hg/HgO electrode (1 M KOH) were used as the reference electrode. The preparation of the working electrodes containing the examined electrocatalysts can be found as follows. A suspension was prepared by dispersing as-prepared catalysts (2.5 mg) and carbon black (0.5 mg, Ketjen Black-300J) in a 0.5 ml mixed solvent containing 140 μL ultrapure water, 340 μL ethanol and 20 μL 5 wt.% Dupont Nafion D521 solution. The mixed solution was then ultrasonicated for 0.5 h to obtain a uniform catalyst ink. After that, a certain volume of dispersion was pipetted onto a piece of clean carbon fiber paper and then dried in air. The catalysts loading was determined as 1 mg cm^−2^. N_2_ and O_2_-saturated 0.1 M KOH solutions were chosen as electrolytes for the HER and OER, respectively. The polarization curves were collected at a scan rate of 5 mV s^−1^ with 95% *iR*-correction. Before collecting, several fast cyclic voltammograms (CVs, 500 mV s^−1^) were taken to clean and stabilize the electrocatalyst surface till steady-state was received. The Nyquist plots were performed at frequencies ranging from 100,000 Hz to 0.1 Hz with an amplitude voltage of 5 mV. The overall-water-splitting performances were collected in 0.1 M and 1 M KOH using the Pt_1_/CoHPO catalyst as both anode and cathode in a H-type electrolytic cell separated by an anion-exchange membrane (fumasep, 130 μm) with *iR*-correction. All recorded potentials (E) were converted to the reversible hydrogen electrode (RHE) based on the following equation: E_RHE_ = E_Hg/HgO_ + 0.059 pH + E^0^_Hg/HgO_. The potential of reference electrode and the pH value of the used electrolyte were examined before the electrochemical measurement (Supplementary Fig. 17).

The turnover frequency (TOF, s^−1^) per metal site were calculated according to the following equation^33,34^:

1 $${{{{{\rm{TOF}}}}}}=j/(n\times F\times N)$$

where *j* is the geometric current density after 95% *iR*-correction, *n* is the number of electrons transferred in the reactions (4 for OER and 2 for HER), *F* is the Faraday constant, and *N* is the molar number of active sites. The number of active sites was calculated on the basis of the hypothesis that all single-atoms in M_1_/CoHPO acted as active centers and neglected the contribution of the CoHPO support^4,5,15^. Specifically, the active site number *N* was estimated via the total catalyst mass loading on the electrode (*m*, mg cm^−2^), the weight percent of active metals in the catalysts (*w*, wt.%, determined by the ICP measurement) and the molar mass (*M*, g mol^−1^) according to the following equation:

2 $$N=m\times w/M$$

Mass activity (MA, A g^−1^) was estimated *via* the catalyst loading (*m*, mg cm^−2^), weight percent of metal in the catalysts (*w*, wt.%) and the geometric current density (*j*, mA cm^−2^) under a certain overpotential based on the following equation:

3 $${{{{{\rm{MA}}}}}}=j/(m\times w)$$

### Ex situ XAFS experiments and data processing

The XAFS spectra data (Pt L_3_-edge, Ru, and Ni K-edges) were performed at the 1W1B beamline of Beijing Synchrotron Radiation Facility (BSRF, 2.5 GeV, a maximum current of 250 mA, Si (311) double-crystal). The ATHENA module of the IFEFFIT software packages was applied to process the acquired XAFS raw data (background subtraction, normalization and Fourier transformation) according to standard procedures^35^. Least-squares curve-fitting of EXAFS data was performed using an ARTEMIS program^36^. Fourier transformation of *k*^3^-weighted EXAFS oscillations, *k*^*3*^χ(k), from k space to *R* space was performed to obtain a radial distribution function. The data were fitted in *R*-space with theoretical models constructed based on the crystal structure derived from DFT optimization. Many-body amplitude-reduction factor (*S*_*0*_^2^) was fixed to 0.787 as determined from Pt foil fitting. Fitting range for Pt_1_/CoHPO was 3 ≤ *k* (/Å) ≤ 10.5 and 1.2 ≤ *R* (Å) ≤ 4.0.

### *Operando* XAFS experiments

The Pt (11,564 eV) L_3_-edge and Co (7,709 eV) K-edge XAFS data were collected at the BL11B station in the Shanghai Synchrotron Radiation Facility (SSRF). A Si (111) double-crystal monochromator was utilized with the energy calibrated using Pt and Co foils. The *operando* XAFS experiment was conducted in a sensitive fluorescence mode using a homemade electrochemical cell and a computer-controlled electrochemical analyzer, and all the data were collected during one period of beam time. An as-prepared Pt_1_/CoHPO/thin carbon paper as the working electrode was in contact with a slip of copper tape and fixed with kapton tape to the exterior of the wall of the cell, with the Pt_1_/CoHPO layer facing inwards. A Pt foil and a Hg/HgO electrode were used as counter electrode and reference electrode, respectively, and a 0.1 M KOH solution was used as the electrolyte.

### *Operando* ATR-FTIR measurements

Electrochemical *operando* ATR-FTIR spectroscopy was performed on a Bruker 70 V FTIR spectrometer with an attenuated-toal-reflection (ATR) configuration using a liquid nitrogen cooled MCT detector. Based on a specially-made three-electrode thin-layer FTIR cell configuration, a FITR radiation sequentially passed by the CaF_2_ window and thin-layer solutions, and then it was reflected by the electrode surfaces. Before each FTIR data collection, the 0.1 M KOH electrolyte was saturated with O_2_ gas, and several fast cyclic voltammograms (500 mV s^−1^) between 0.05 and 1.35 V *versus* RHE were first taken to obtain a stable current response. The FTIR spectra were collected at an open-circuit voltage, and a potential range of 1.0-1.6 V *versus* RHE with an interval of 0.1 V. Each infrared absorption spectrum was acquired at a resolution of 4 cm^−1^.

### MEA fabrication and evaluation

Pt_1_/CoHPO catalyst was integrated into a MEA to assemble an AEMWE device, and evaluated its electrochemical performance using an 850e fuel cell test system equipped with 885-HS potentiostat. Alkymer (Ionomr Innovations Inc.) was used as the anion exchange membrane (AEM) and immersed in 1 M KOH solution for at least 24 h prior to being used to exchange Cl^−^ into OH^−^. To prepare the catalyst ink, 20 mg of Pt_1_/CoHPO catalyst and 2 mg of Ketjen Black were homogeneously dispensed into 6 mL of mixed solvent containing ultrapure water, ethanol and Dupont D521 under sonication for about 1 h at room temperature. Both anodic and cathodic electrodes were fabricated by the catalyst-coated membrane (CCM) method. Specifically, membrane pieces were placed and taped flat onto a hotplate (P-2030, Polish) with an exposed membrane surface area of 1.0 cm^2^. The temperature of the hotplate was 80 °C, ensuring that the solvent evaporated quickly. In addition, vacuum was also used to confirm the membrane firmly adsorbed on the hotplate. The well dispersed catalyst ink was then sprayed with a spray gun (Mr. Hobby, Japan) until the loading was ~2 mg cm^−2^ (11.4 μg_Pt_ cm^−2^) for cathode. For the anodic electrode, the catalytic ink was coated on the opposite membrane side by the same process described above with the loading of ~3 mg cm^−2^ (17.1 μg_Pt_ cm^−2^). Commercial IrO_*x*_ and Pt/C electrodes were also prepared in the above method using Ir oxide (Alfa Aesar) and Pt/C (20 wt.% Pt, Alfa Aesar), respectively. At the end, the as-prepared MEA was assembled into a homemade integrated AEMWEs device with titanium mesh and carbon paper (GDL-29BC, Sigracet) as the anodic and cathodic gas diffusion layer, respectively. Electrically insulating gaskets were also placed to prevent the liquid and gas from escaping through any space between flow fields. The torque applied when assembling the cell was ~5 Nm. 0.1 M KOH was circulated through the anodic side by a peristaltic pump. Polarization curves were collected from 1.0 V to 2.2 V at a water temperature from 25 °C to 80 °C under an ambient pressure. A stability test was carried out by potentiostatic electrolysis at a constant cell voltage of 1.9 V at about 30 °C. Before the stability curves were obtained, a potentiostatic electrolysis was held for 20 min to reach a steady state.

### DFT calculations

DFT calculations were performed using the Vienna ab initio Simulation Package (VASP) with the projector-augmented wave (PAW) pseudopotentials^37,38^. The exchange-correlation interaction was described by using the Perdew-Burke-Ernzerhof (PBE) functional^39^. The plane-wave energy cut-off was taken as 400 eV. The Brillouin zone was sampled based on the Monkhorst-Pack scheme with a 3 × 3 × 1 k-point mesh^40^. The Pt_1_/CoHPO slab model was constructed based on the experimental results of the EXAFS spectra (Supplementary Fig. 7). The model structure was further examined by calculation of the XANES spectra of the Pt atoms using FEFF8 (Supplementary Fig. 9)^41^. The adjacent slabs were separated by a 15 Å vacuum in the normal direction. The contributions of zero-point energy, entropic and solvent corrections to the free energies of the reaction intermediates were considered^42^. The free energy change in each reaction step was calculated based on the computational hydrogen electrode (CHE) model^32^. In particular, the free energy of an electron-proton pair in the CHE model is computed as a half of the free energy of H_2_ molecule at the standard conditions, which is then shifted by –eU upon an applied external potential U.

## Supplementary information

Supplementary Information

## Data Availability

The data supporting the findings of this study are available within the article and its Supplementary Information. Source data are provided with this paper.

## Source data

Source Data
