# Author Correction: Anti-dissolution Pt single site with Pt(OH)(O_3_)/Co(P) coordination for efficient alkaline water splitting electrolyzer

**DOI:** 10.1038/s41467-022-31988-9

**Published:** 2022-07-25

**Authors:** Lingyou Zeng, Zhonglong Zhao, Fan Lv, Zhonghong Xia, Shi-Yu Lu, Jiong Li, Kaian Sun, Kai Wang, Yingjun Sun, Qizheng Huang, Yan Chen, Qinghua Zhang, Lin Gu, Gang Lu, Shaojun Guo

**Affiliations:** 1 School of Materials Science and Engineering, Peking University, Beijing, China; 2 School of Physical Science and Technology, Inner Mongolia University, Hohhot, China; 3 Shanghai Synchrotron Radiation Facilities, Shanghai Institute of Applied Physics, Chinese Academy of Science, Shanghai, China; 4 Department of Chemistry, Tsinghua University, Beijing, China; 5 Beijing National Laboratory for Condensed Matter Physics, Institute of Physics, Chinese Academy of Sciences, Beijing, China; 6 Department of Physics and Astronomy, California State University Northridge, Northridge, CA USA

**Keywords:** Electrocatalysis, Electrocatalysis, Nanoscale materials

Correction to: *Nature Communications* 10.1038/s41467-022-31406-0, published online 02 July 2022.

Due to an error, the original version of this Article incorrectly listed the references. In the tenth line of the second paragraph of main article, the references 18, 20, and 21 were previously incorrectly cited as reference 19, 21, and 22.

In the fifth line of the third paragraph of the main article, References 1, 21 were previously incorrectly cited as references 1, 23.

In the twenty-third line of the second paragraph in the Results section, Reference 22 was previously incorrectly cited as reference 24.

In the fifteenth line of the third paragraph in the Results section, Reference 23 was previously incorrectly cited as reference 25.

In the eleventh line of the eighth paragraph of the Results section, References 24, 25 were previously incorrectly cited as references 26, 27.

In the seventeenth line of the eighth paragraph of the Results section, Reference 26 was previously incorrectly cited as reference 28.

In the twenty-fourth line of the eighth paragraph of the Results section, References 27, 28 were previously incorrectly cited as references 29, 30.

In the twenty-seventh line of the eighth paragraph of the Results section, Reference 29 was previously incorrectly cited as reference 31.

In the twenty-third line of the tenth paragraph of the Results section, References 30, 31 were previously incorrectly cited as references 32, 33.

In the third line of the eleventh paragraph of the Results section, Reference 32 was previously incorrectly cited as reference 34.

In the second line of the fourth paragraph of the Methods section, References 33, 34 were previously incorrectly cited as references 35, 36.

In the sixth line of the sixth paragraph of the Methods section, Reference 35 was previously incorrectly cited as reference 37.

In the seventh line of the sixth paragraph of the Methods section, Reference 36 was previously incorrectly cited as reference 38.

These have been corrected in both the PDF and HTML versions of the Article.

