# Supplementary material for: Anti-dissolution Pt single site with Pt(OH)(O3)/Co(P) coordination for efficient alkaline water splitting electrolyzer
Source: Nat Commun. 2022 Jul 2;13:3822. doi: 10.1038/s41467-022-31406-0 (PMC9250493; doi:10.1038/s41467-022-31406-0)
Supplement: Supplementary file 1 — Supplementary Information [file 41467_2022_31406_MOESM1_ESM.pdf]

Supplementary Information for

Anti-dissolution Pt single site with $Pt(OH)(O_3)/Co(P)$ coordination for efficient alkaline water splitting electrolyzer

Lingyou Zeng[1,+], Zhonglong Zhao[2,+], Fan Lv[1], Zhonghong Xia[1], Shi-Yu Lu[1], Jiong Li[3], Kaian Sun[4], Kai Wang[1], Yingjun Sun[1], Qizheng Huang[1], Yan Chen[1], Qinghua Zhang[5], Lin Gu[5], Gang Lu[6*] and Shaojun Guo[1*]

[1]School of Materials Science and Engineering, Peking University, Beijing, China.
[2]School of Physical Science and Technology, Inner Mongolia University, Hohhot, China.
[3]Shanghai Synchrotron Radiation Facilities, Shanghai Institute of Applied Physics, Chinese Academy of Science, Shanghai, China
[4]Department of Chemistry, Tsinghua University, Beijing, China.
[5]Beijing National Laboratory for Condensed Matter Physics, Institute of Physics, Chinese Academy of Sciences, Beijing, China.
[6]Department of Physics and Astronomy, California State University Northridge, Northridge CA, USA.

[+]These authors contributed equally: Lingyou Zeng, Zhonglong Zhao

*Correspondence author. Email: ganglu@csun.edu (G. L.); guosj@pku.edu.cn (S. G.)

Supplementary methods

Chemicals. Hexamethylenetetramine (HMT), NaCl, $CoCl_2 \cdot 6H_2O$, Na_2HPO_4, $NiCl_2 \cdot 6H_2O$, $FeCl_2 \cdot 4H_2O$, $RuCl_3 \cdot xH_2O$, K_2PtCl_6 and $IrCl_3 \cdot xH_2O$ were purchased from Aladdin Reagent Co. Nafion solution (Dupont, 5 wt.% polymer content) was obtained from Alfa Aesar. 20 wt.% platinum/iridium on activated carbon were purchased from Johnson-Matthey Corporation.

Preparation of CoHPO support. The CoHPO support was prepared *via* an anion exchange reaction between α-$Co(OH)_2$ and HPO_4^{2-}. To synthesize α-$Co(OH)_2$ precursor[1], $CoCl_2 \cdot 6H_2O$, NaCl, HMT were simultaneously dissolved in a mixed solution of 180 mL deionized water and 20 mL ethanol to obtain the final concentrations of 10, 50 and 60 mM, respectively, and then heated at 90 °C for 1 h under stirring condition. To obtain CoHPO, 0.1 g of as-prepared α-$Co(OH)_2$ was dispersed in a 100 mL beaker of 1 M Na_2HPO_4 aqueous solutions. The suspension was then stirred at 30 °C for 24 h. The resulting precipitate was collected by centrifugation, and dried at 60 °C overnight.

Preparation of Pt_{NP}/CoHPO. To prepare Pt nanoparticles on the CoHPO, 30 mg CoHPO and 0.1 mL of 10 mg mL^{-1} K_2PtCl_6 were added into 60 mL ethylene glycol, and then continuously sonicated for 3 h. After that, excess amount of 0.1 M $NaBH_4$ was dropwise into the dispersion under continuous stirring. After stirred for 1 h, the precipitate was centrifugalized and washed *via* ethanol for three times, and dried in an oven overnight and reduced in 10% H_2/Ar at 110 °C for 90 min.

Materials characterization. TEM images were conducted on a HITACHI H-7700 operating at an accelerating voltage of 100 kV. HAADF-STEM and corresponding elemental analysis were taken on JEM ARM200F TEM instruments. XRD was performed on a PANalytical instrument equipped with a Cu Kα radiation. XPS spectra were carried out on Thermo Fisher Scientific instrument (Escalab 250Xi). The content of metals in the catalysts was determined by ICP-AES on an Agilent 8800 instrument.

Supplementary figures and tables

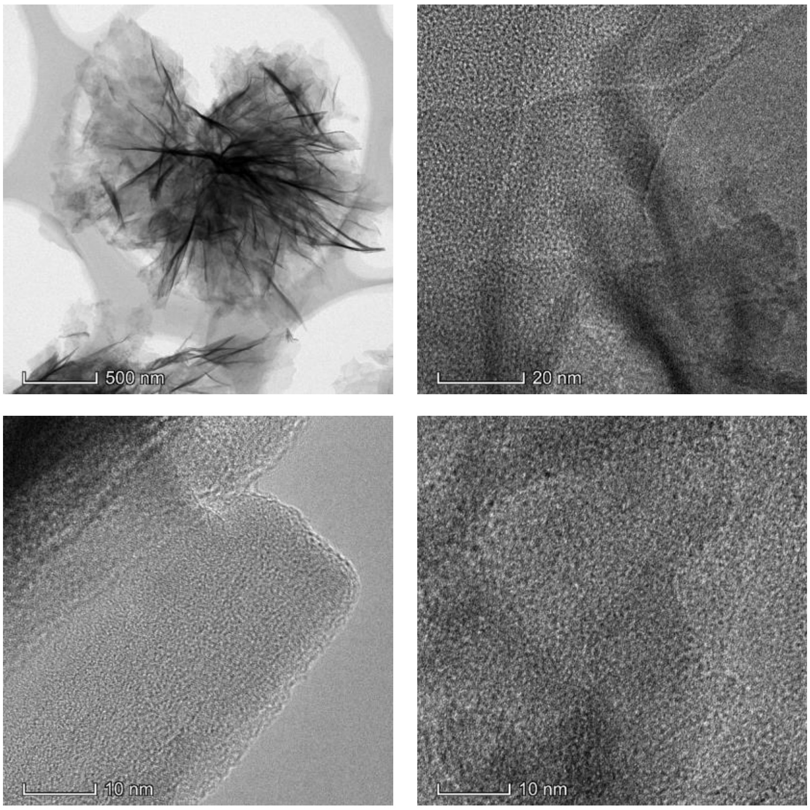

Supplementary Fig. 1 | TEM images of CoHPO at different magnifications. Low-resolution TEM image reveals a microflower-like morphology with a diameter of ~1.0 μm formed by aggregated ultrathin nanosheets or flakes, and the high-resolution TEM images prove the amorphous nature of each nanosheet or flake, which provides abundant defect sites that adsorb and anchor the atomically dispersed metal atoms, and prevent their migration and aggregation.

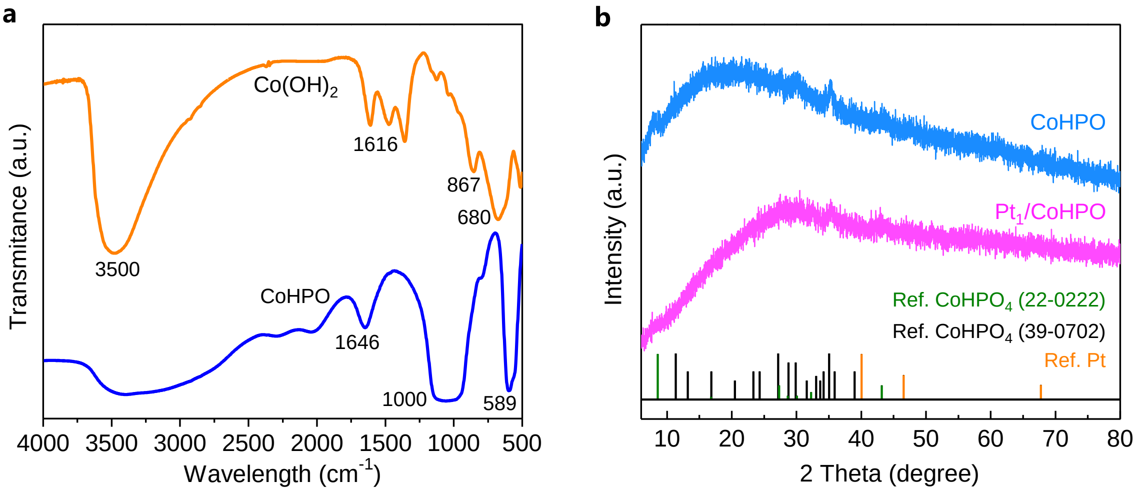

Supplementary Fig. 2 | Compositional characterization. (a) FTIR spectra of α-$Co(OH)_2$ precursor and CoHPO. (b) XRD patterns of CoHPO and Pt_1/CoHPO.

For the FTIR spectrum of α-$Co(OH)_2$, the wide band at ~680 cm^{-1} is associated with Co-O stretching and Co-OH bending vibrations, and the weak band at ~867 cm^{-1} is the characteristic of carbonate ions[1]. Meanwhile, the large bands centered at ~3500 cm^{-1} and the peak at ~1616 cm^{-1} are assigned to the O-H stretching mode and bending mode of the interlayer water molecules, respectively. For the CoHPO sample, the wide peak centered at ~1050 cm^{-1} belongs to the stretching vibration of P-OH, and a sharp peak appearing at ~589 cm^{-1} can be assigned to the P-OH wagging vibrational modes, which validate the formation of the $CoHPO_4$[2,3]. Furthermore, the XRD pattern in Supplementary Fig. 2b reveals its amorphous nature of CoHPO, with weak diffraction peaks assigned to the $CoHPO_4$.

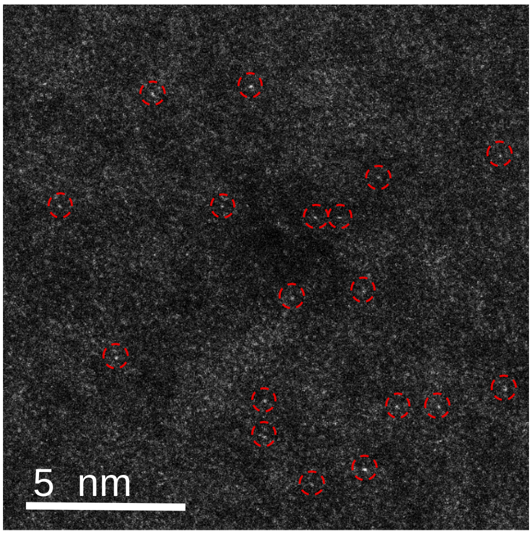

Supplementary Fig. 3 | Additional aberration-corrected HADDF-STEM images of Pt_1/CoHPO catalysts. The red and white circles represent single-site Pt.

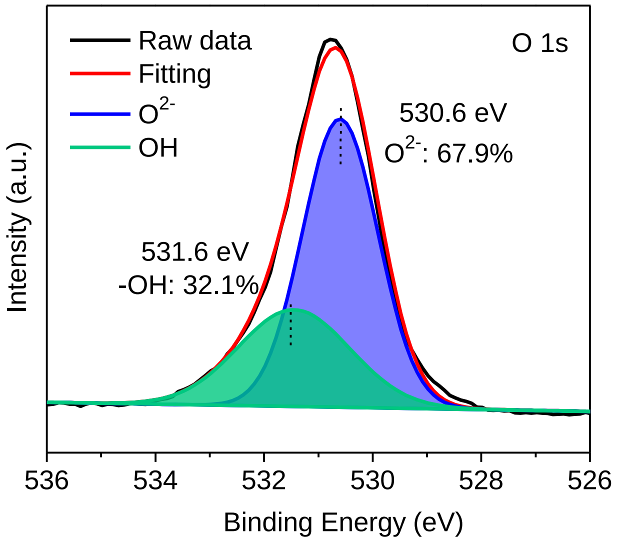

Supplementary Fig. 4 | O 1s XPS spectra of the Pt_1/CoHPO catalyst. The O 1s XPS spectra display two peaks with one major peak at ~530.6 eV and a shoulder peak centered at ~531.6 eV, corresponding to the existence of O^{2-} (ratio: 67.9%) and OH^- (ratio: 32.1%), respectively.

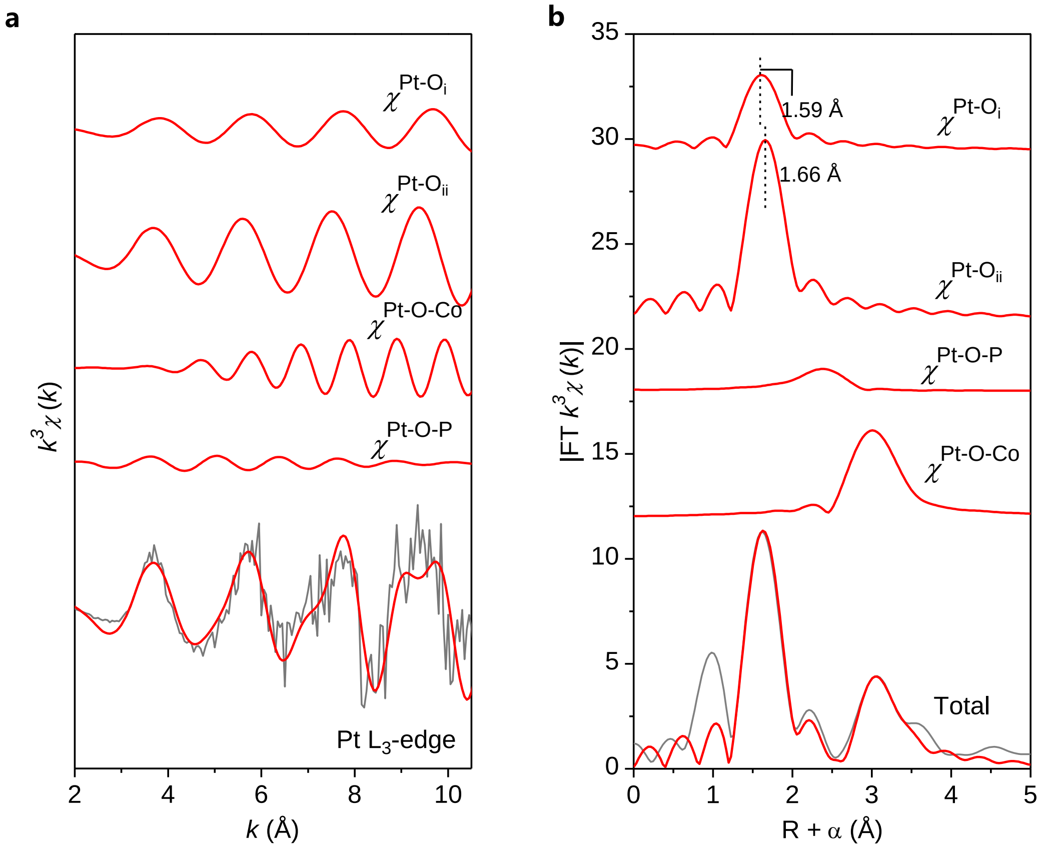

Supplementary Fig. 5 | Pt L_3-edge EXAFS analysis of Pt_1/CoHPO in (a) *k* spaces and (b) *R* spaces. The curves from top to bottom are the Pt-O_i, Pt-O_{ii}, Pt-O-Co and Pt-O-P scattering signals included in the fit and the total signal (red line) superimposed on the experimental data (gray line). Measured and fitted spectra are well matched. The detailed fit parameters are shown in Supplementary Table 1.

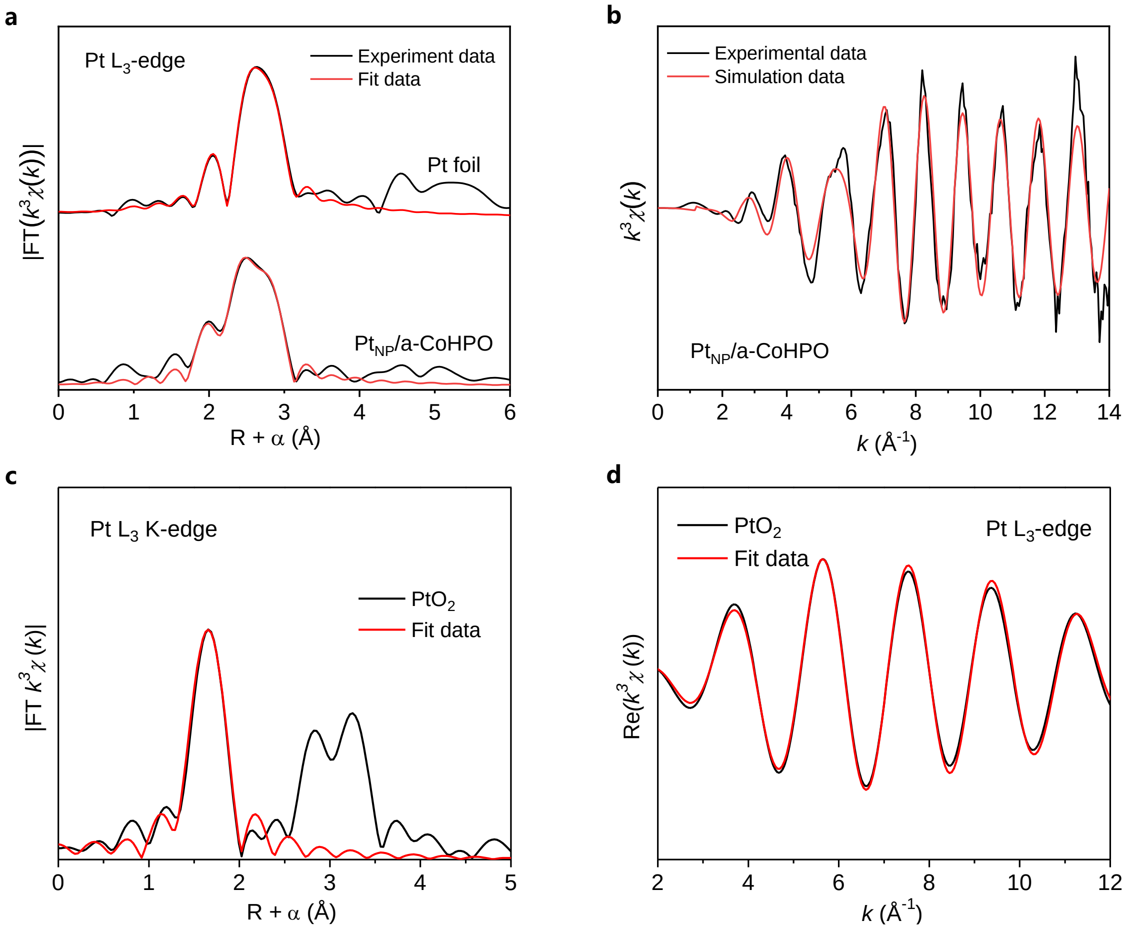

Supplementary Fig. 6 | Fourier-transformed magnitude of EXAFS spectra in *k* space and *R* space. (a) Pt foil, (b) Pt_{NP}/CoHPO and (c, d) PtO_2. Measured and fitted spectra are well matched for all samples. The detailed fit parameters are shown in Supplementary Table 1.

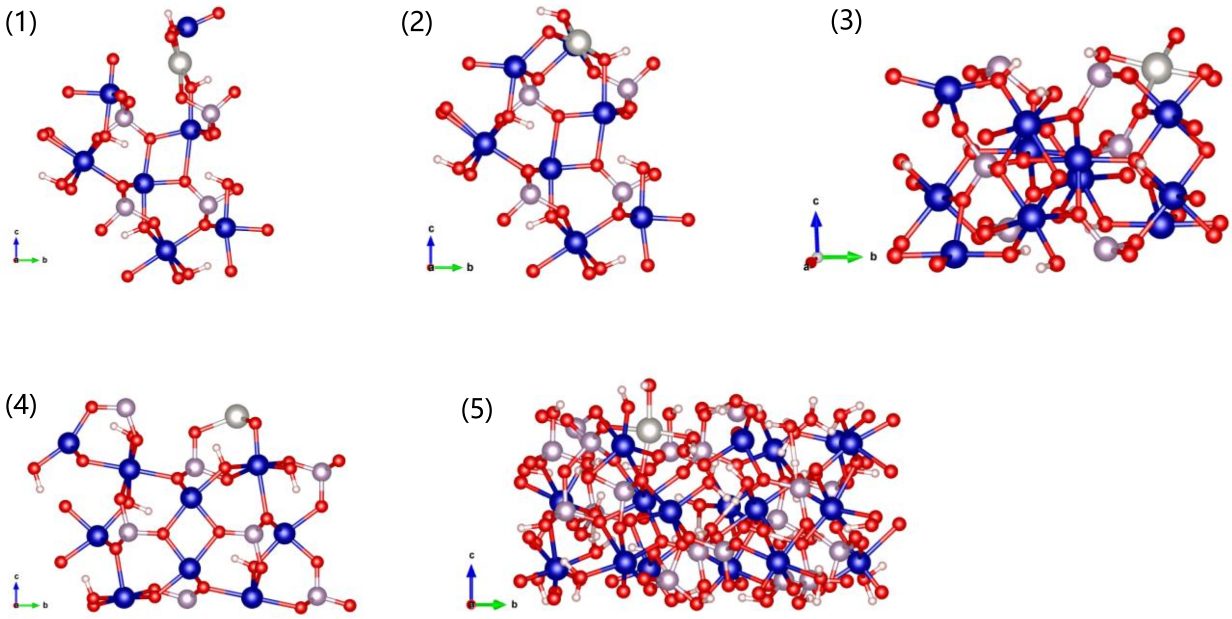

Supplementary Fig. 7 | The 5 possible structural model of Pt_1/CoHPO with Pt(OH)(O)/Co(P) coordination embedded in CoHPO surface. The numbers (1) to (5) represent the model-1 to model-5. Pt (gray), Co (blue), O (red), P (pink) and H (white).

Given that the cobalt hydrogen phosphate (CoHPO) is an amorphous material, 5 possible structural model of Pt_1/CoHPO with Pt(OH)(O)/Co(P) coordination embedded in CoHPO surface were built. These optimized atomic structures and corresponding structure parameters (Pt-O/OH bond length) are shown in Supplementary Fig. 7 and Supplementary Table 2, respectively. It is found that the optimized bond lengths of Pt-O and Pt-OH of model-3 are in good agreement with the experimental EXAFS data (Supplementary Fig. 8 and Table 1). Moreover, the model structure was further examined by calculation of the Pt L_3-edge XANES spectra, which has high sensitivity to the three-dimensional arrangement of atoms[4]. The simulated XANES spectrum based on model-3 match excellently with the experimental spectra (Supplementary Fig. 9), further demonstrating that the structural model-3 used in the DFT calculation is reasonable.

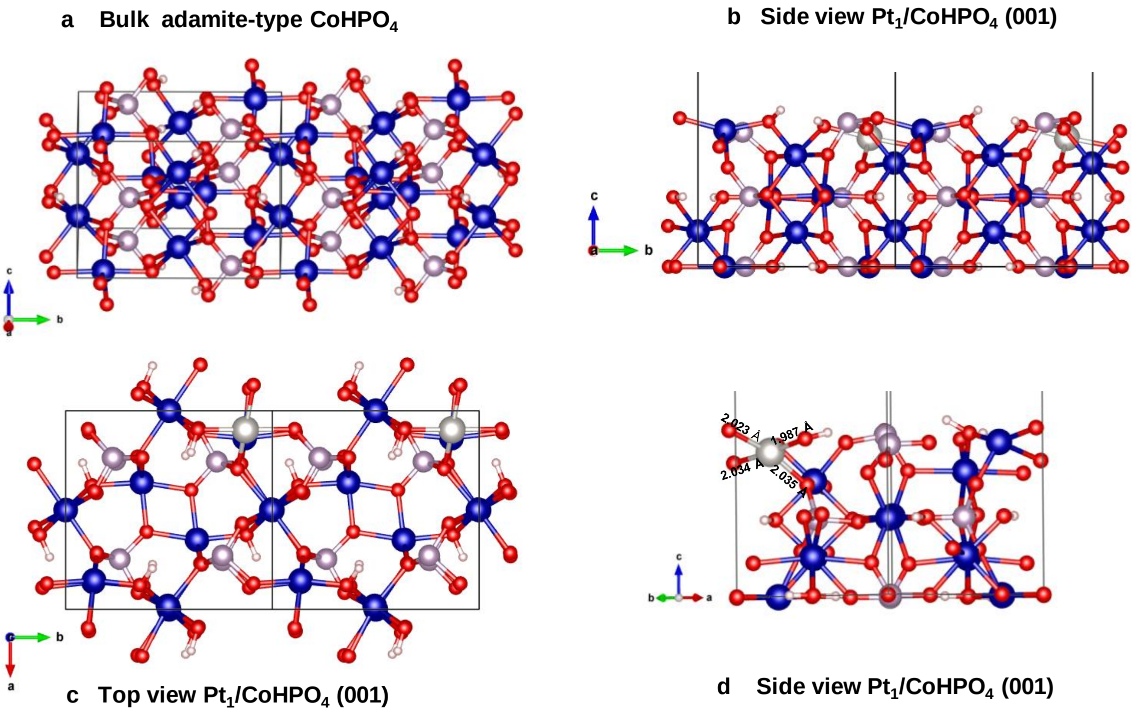

Supplementary Fig. 8 | Slab models of the $CoHPO_4$ and $Pt_1/CoHPO_4$ structure. Pt (gray), Co (blue), O (red), P (pink) and H (white). (a) Bulk adamite-type $CoHPO_4$. (b) side view of $Pt_1/CoHPO_4$ (001) surface. (c) Top view of $Pt_1/CoHPO_4$ (001) surface. (d) Side view of $Pt_1/CoHPO_4$ (001) surface. The calculated average bond length of Pt-O bond and Pt-OH bond is 2.031 Å and 1.987 Å, respectively, which is in good agreement with the EXAFS results (Supplementary Table 1).

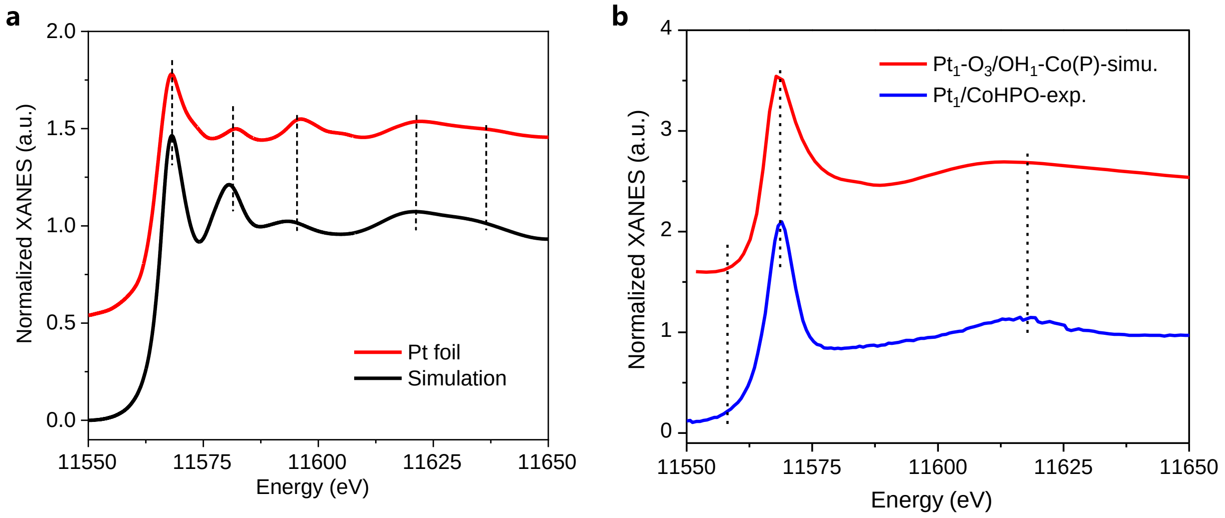

Supplementary Fig. 9 | The simulated and experimental XANES spectrum. (a) The Pt foil. (b) The simulated Pt L_3-edge spectrum based on the structure of model-3 (Supplementary Fig. 8) and experimental Pt L_3-edge spectrum of Pt_1/CoHPO.

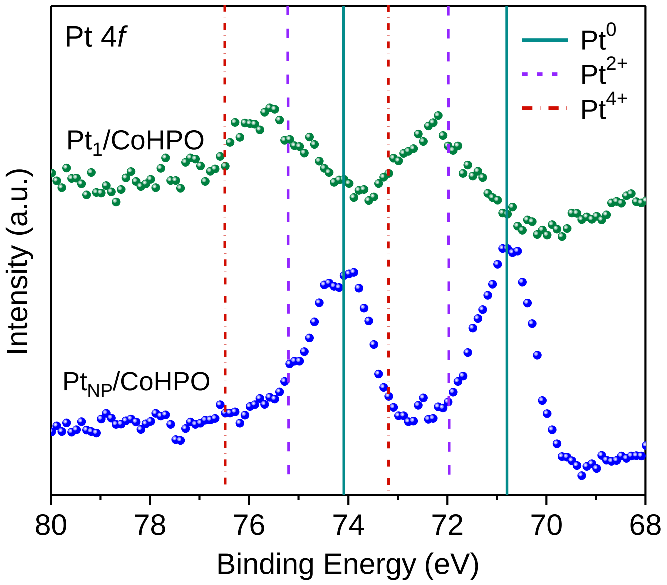

Supplementary Fig. 10 | XPS spectra of Pt_1/CoHPO and Pt_{NP}/CoHPO for Pt 4*f* regions. The Pt 4*f* binding energy of Pt_1/CoHPO positively shifts compared to that of Pt nanoparticles/CoHPO, locating between those of Pt^{2+} and Pt^{4+}. This result reveals that the Pt center in Pt_1/CoHPO has partial positive charges, with a valence state between Pt^0 to Pt^{4+}.

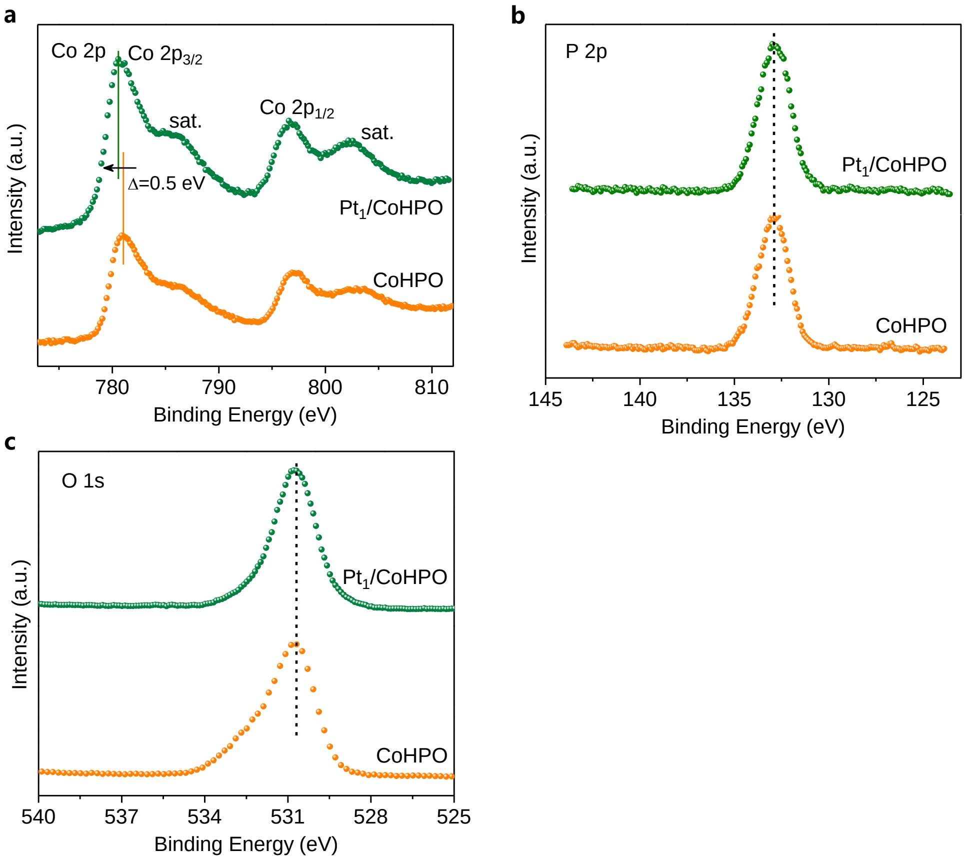

Supplementary Fig. 11 | XPS spectra of Pt_1/CoHPO and CoHPO. (a) Co 2*p* spectra. (b) P 2*p* spectra. (c) O 1*s* spectra. The binding energy of Co 2*p* (Supplementary Fig. 11a) in Pt_1/CoHPO has an obviously negative shift, whereas that of P 2*p* (Supplementary Fig. 11b) and O 1*s* (Supplementary Fig. 11c) was unchanged relative to that of CoHPO support, implying that the electron transfer from CoHPO to Pt depends on the Co-O-Pt bonds rather than the P-O-Pt bonds.

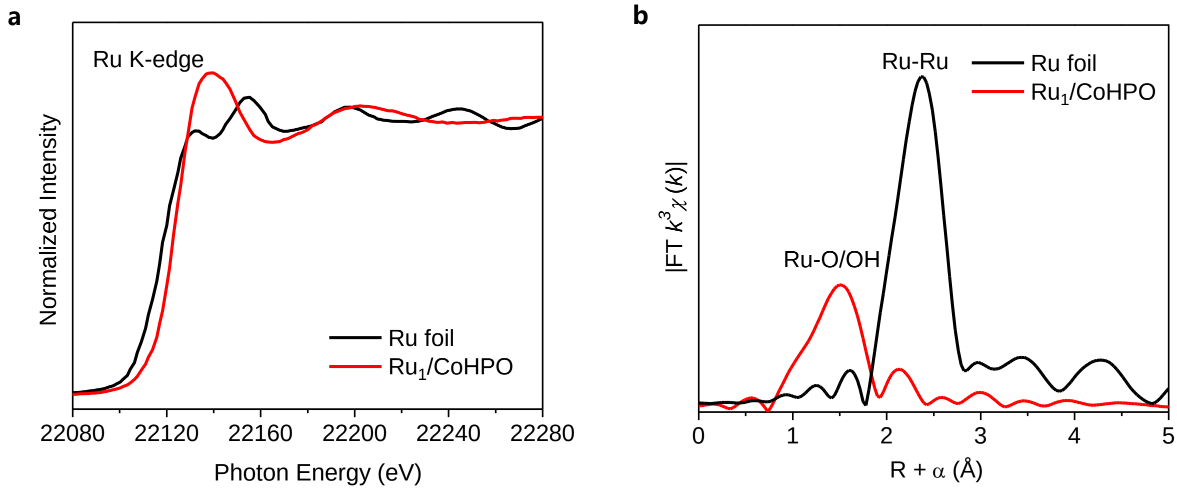

Supplementary Fig. 12 | X-ray absorption analyses of Ru_1/CoHPO. (a) Ru K-edge XANES spectra. (b) FT-EXAFS spectra. As shown in Supplementary Fig. 12a, the while line for Ru_1/CoHPO exhibits different peak positions from Ru foil, indicating diverse configurations of Ru species in Ru_1/CoHPO. The FT-EXAFS curves of Ru_1/CoHPO (Supplementary Fig. 12b) shows a dominant peak at ~1.5 Å, corresponding to the Ru-O/OH coordination. No appreciable Ru-Ru peak at 2.4-3.0 Å is observed in Ru_1/CoHPO, confirming the atomically dispersion of Ru species.

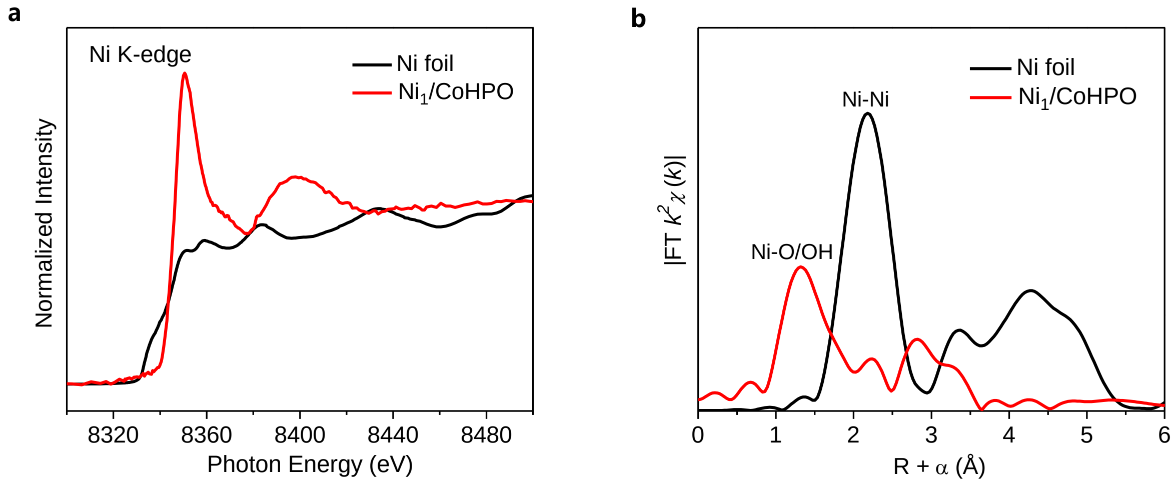

Supplementary Fig. 13 | X-ray absorption analyses of Ni_1/CoHPO. (a) Ni K-edge XANES spectra. (b) FT-EXAFS spectra. The FT-EXAFS curves of Ni_1/CoHPO (Supplementary Fig. 13b) show a dominant peak at ~1.3 Å, corresponding to the Ni-O/OH coordination. No appreciable Ni-Ni peak at 1.8-2.2 Å is observed in Ni_1/CoHPO, confirming that Ni specie is predominantly existed as atomically coordinated Ni-O/OH moieties.

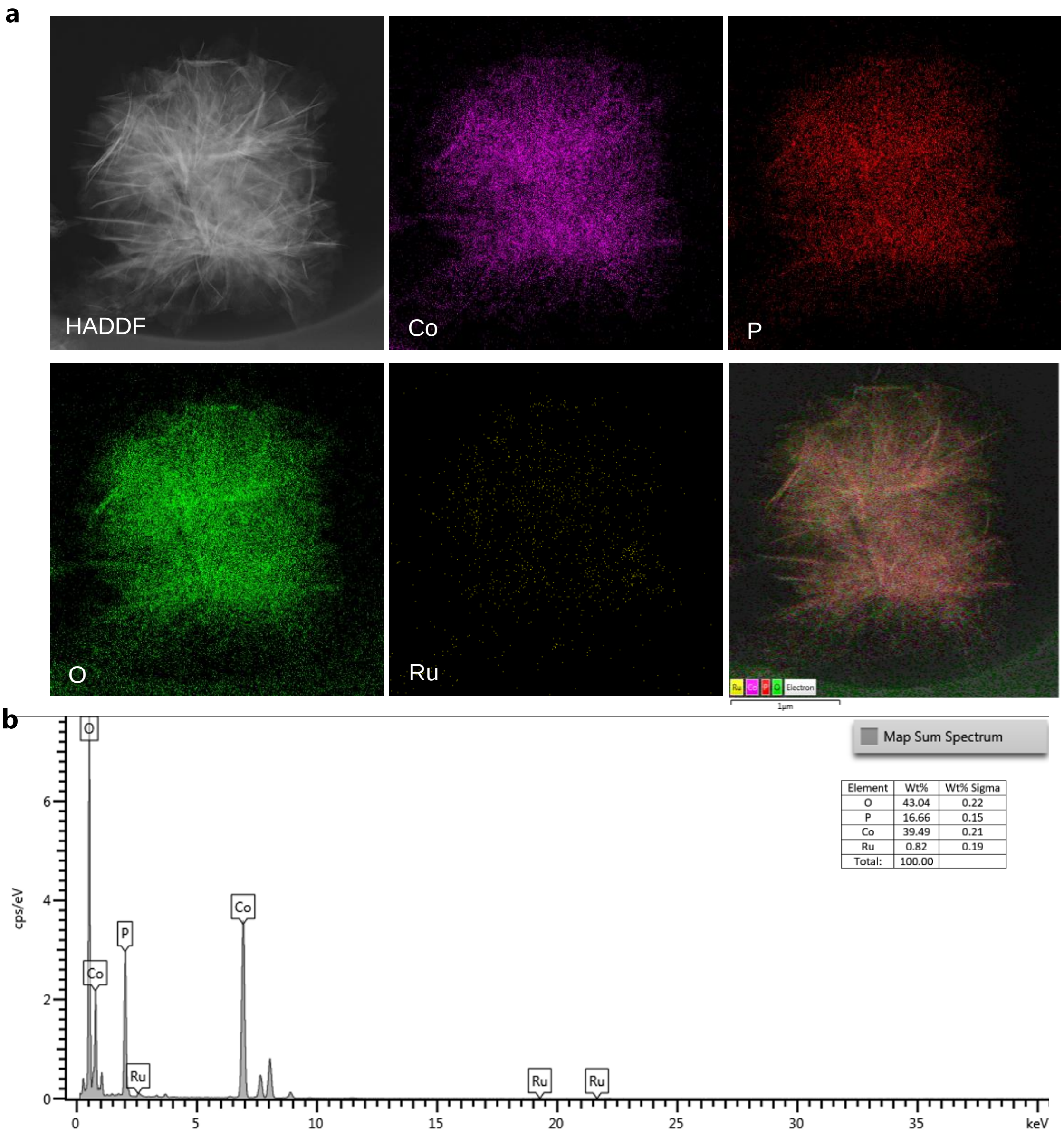

Supplementary Fig. 14 | TEM and EDX characterizations of Ru_1/CoHPO. (a) TEM image and elemental mapping images. (b) EDX spectrum.

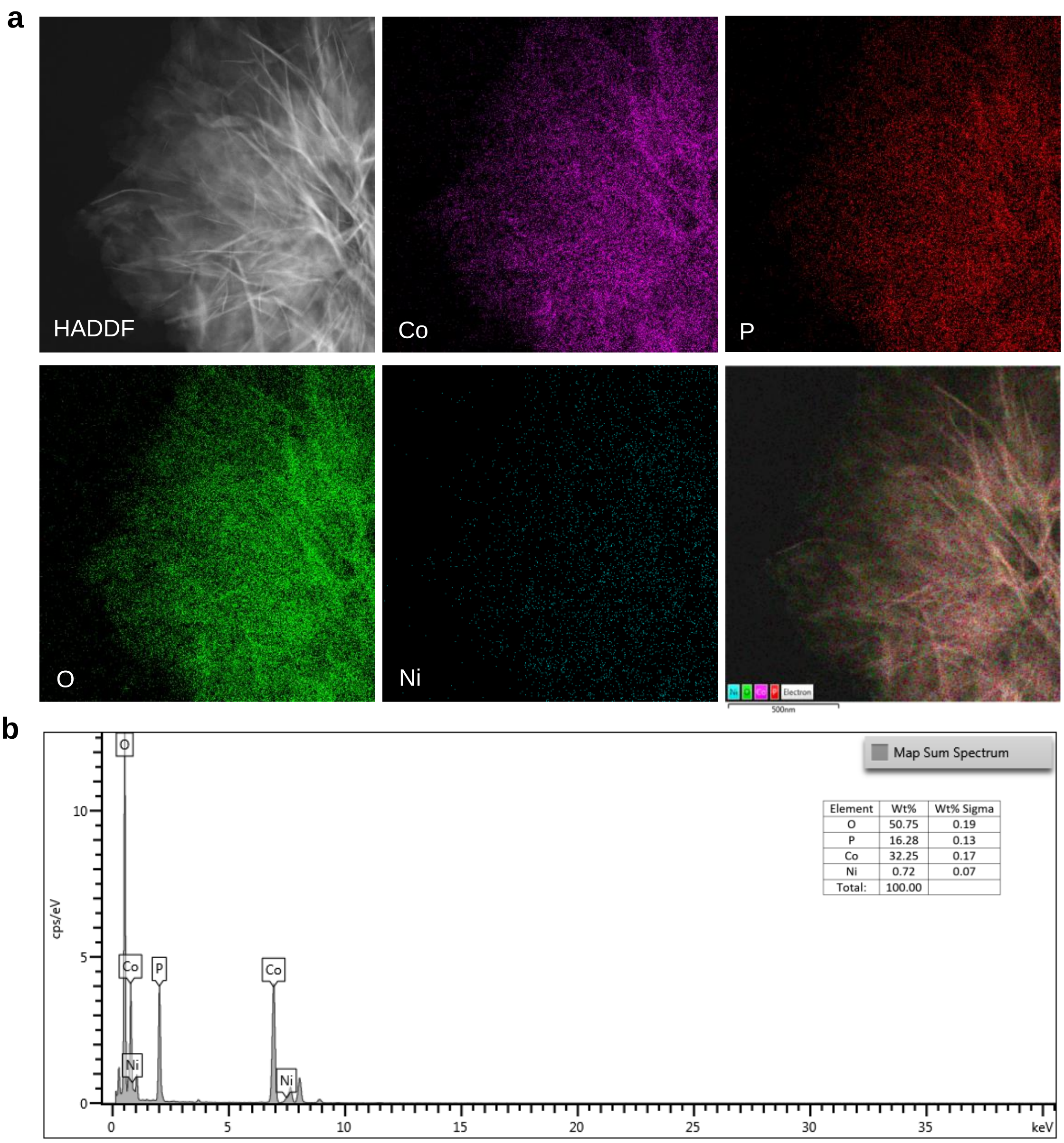

Supplementary Fig. 15 | TEM and EDX characterizations of Ni$_1$/CoHPO. (a) TEM image and elemental mapping images. (b) EDX spectrum.

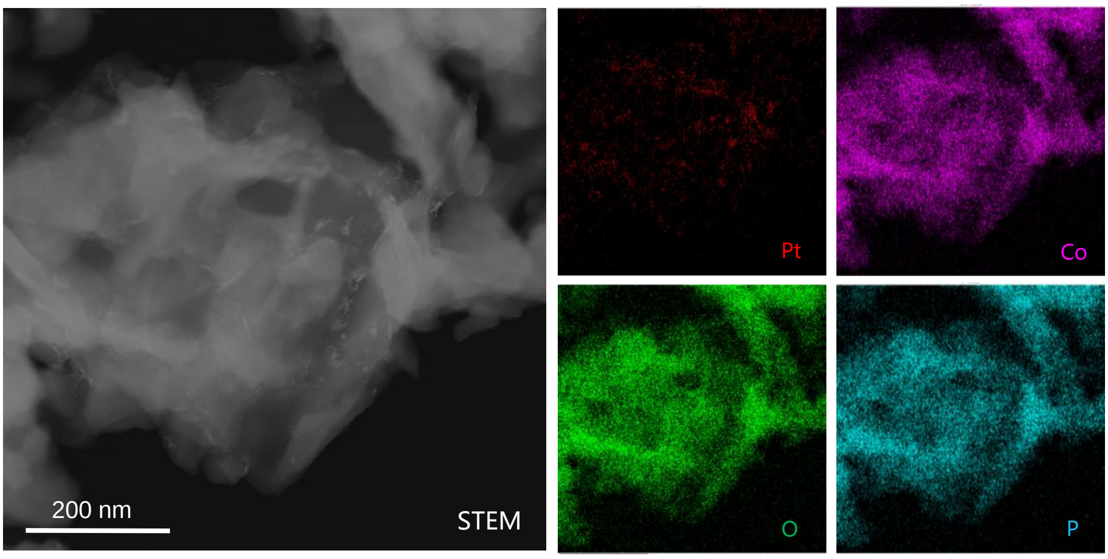

Supplementary Fig. 16 | STEM images and corresponding elemental mapping of Pt_{NP}/CoHPO sample (synthesized by a $NaBH_4$-reduction method).

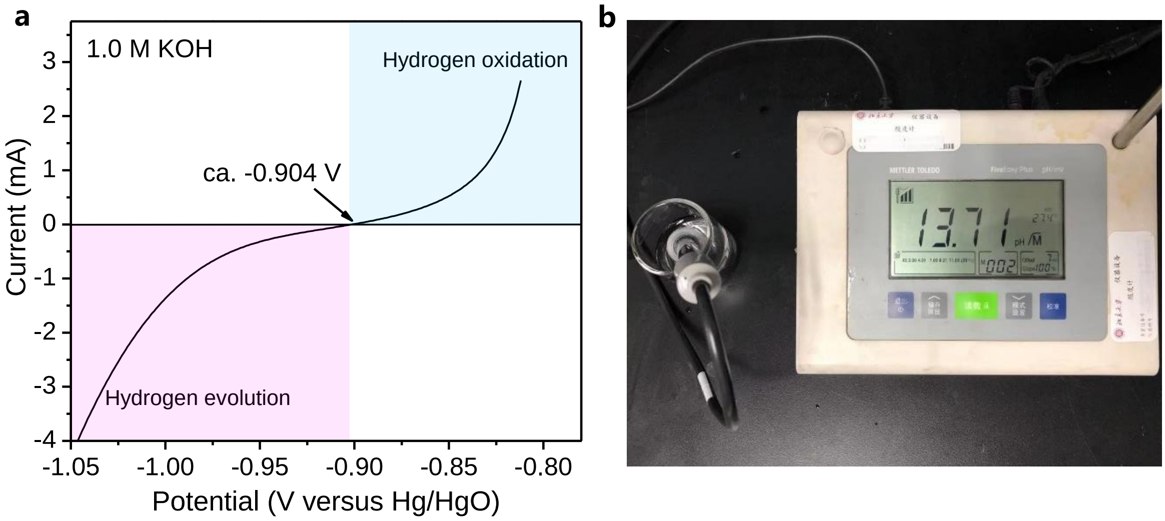

Supplementary Fig. 17 | Electrode calibration and pH measurement of the electrolyte. (a) Calibration of Hg/HgO electrode was carried out in a conventional three-electrode setup with 1 M KOH solution. (b) The pH test of the used 1 M KOH electrolyte. As displayed in Supplementary Fig. 17a, the zero current point is at about -0.904 V in 1 M KOH, so E(RHE) = E(Hg/HgO) + 0.904 V. And, the pH value of the used electrolyte was checked by the pH meter. As shown in Supplementary Fig. 17b, the pH value of the 1 M KOH solution in this work is 13.71. Consequently, the potential of the used Hg/HgO reference electrode is ~0.0951 V, which is very close to the standard potential (0.0977 V, Hg/HgO/25 °C).

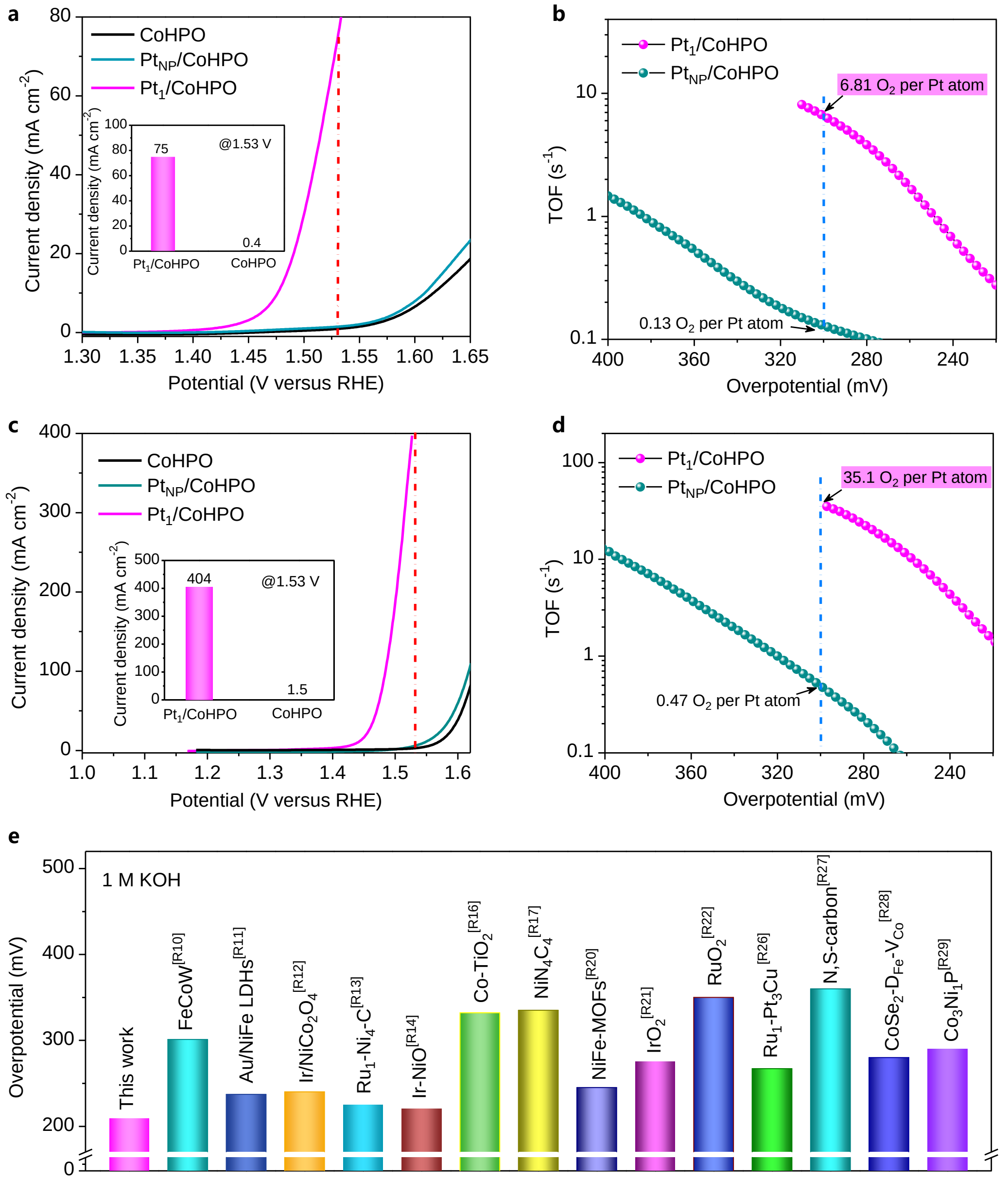

Supplementary Fig. 18 | Oxygen evolution reaction performance in 0.1 M KOH and 1 M KOH solutions. OER polarization curves and TOFs curves of different catalysts at different overpotentials in (a, b) 0.1 M KOH solution and (c, d) 1 M KOH solution. (e) OER overpotential at Pt_1/CoHPO and recently reported electrocatalysts (Supplementary Table 3) at current density of 10 mA cm^{-2}. As shown in Supplementary Fig. 18a and c, Pt_1/CoHPO exhibits current densities of 75 and 404 mA cm^{-2} at overpotential of 300 mV in 0.1 M and 1 M KOH, respectively, two orders of magnitude higher than those of CoHPO support, indicating the rather poor activity of CoHPO in comparison to Pt_1/CoHPO. Moreover, Pt_1/CoHPO exhibits TOF values of 6.81 ± 0.13 and 35.1 ± 5.2 s^{-1} *per* Pt atoms in 0.1 M and 1 M KOH (Supplementary Fig. 17 b and d), respectively, which are more than 50-fold larger than that of nanoparticle Pt_{NP}/CoHPO counterpart (0.13 and 0.47 s^{-1}). All this results strongly suggest that the single atomic Pt_1 is vital to the high OER performance of Pt_1/CoHPO.

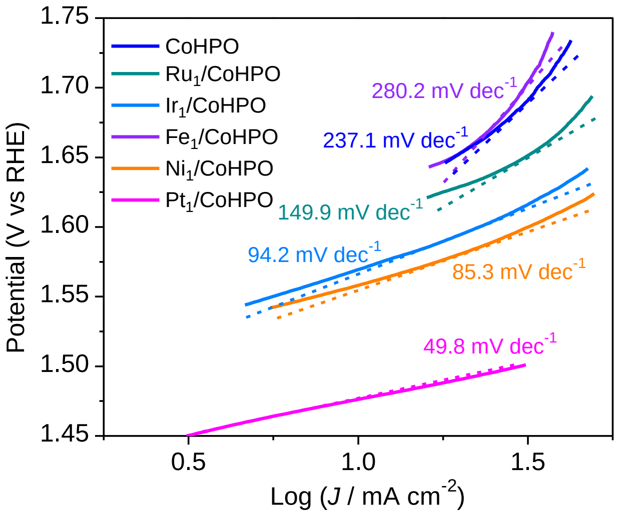

Supplementary Fig. 19 | Tafel slopes of various catalysts in 0.1 M KOH. The Tafel slopes were derived from OER polarization curves.

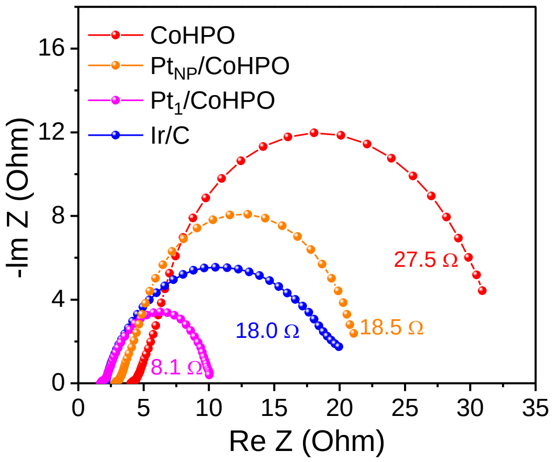

Supplementary Fig. 20 | EIS results of different catalysts. EIS spectra of CoHPO, Pt_{NP}/CoHPO, Pt_1/CoHPO and Ir/C in 0.1 M KOH.

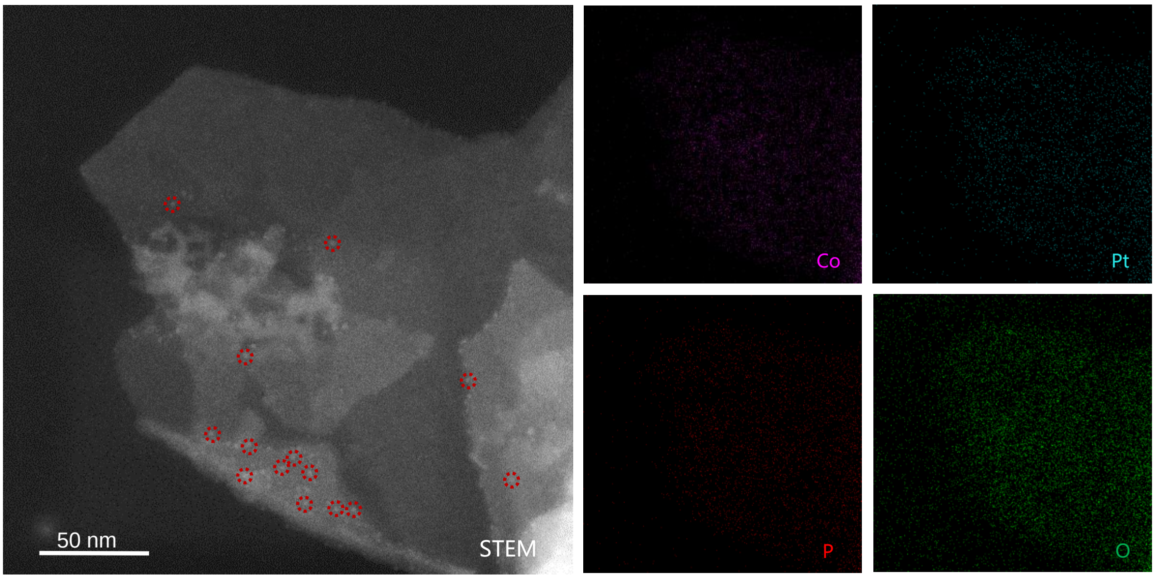

Supplementary Fig. 21 | STEM image and corresponding elemental mapping of the Pt_1Pt_{NP}/CoHPO sample. As shown in Supplementary Fig. 21, some small Pt clusters or nanoparticles (marked by red circles) can be observed in the resulting Pt (0.95 wt.%)/CoHPO catalyst.

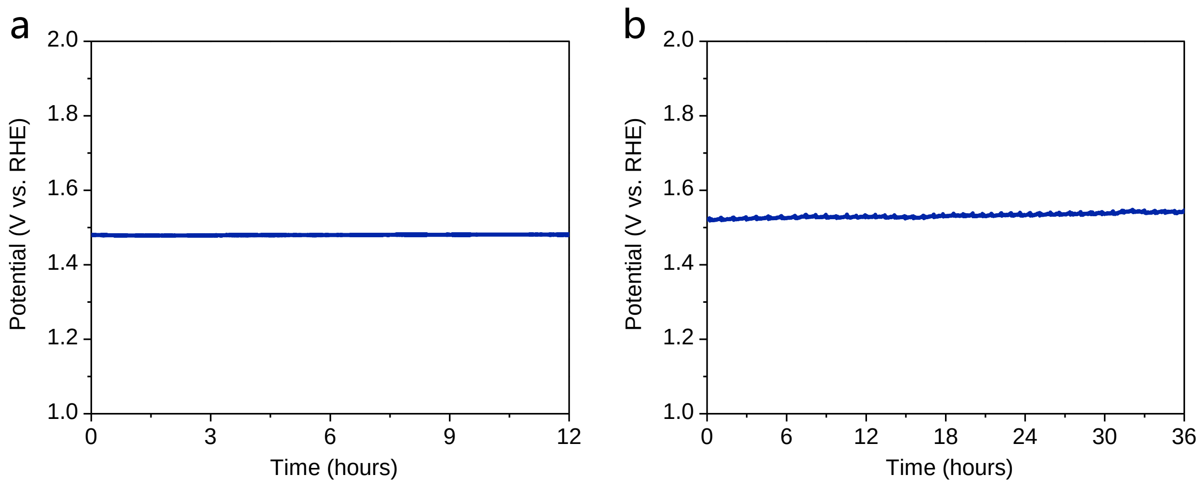

Supplementary Fig. 22 | OER stability tests. The potential-time responses of the Pt_1/CoHPO catalyst at current densities of (a)10 mA cm^{-2} and (b) 100 mA cm^{-2} in a typical three-electrode system for the OER.

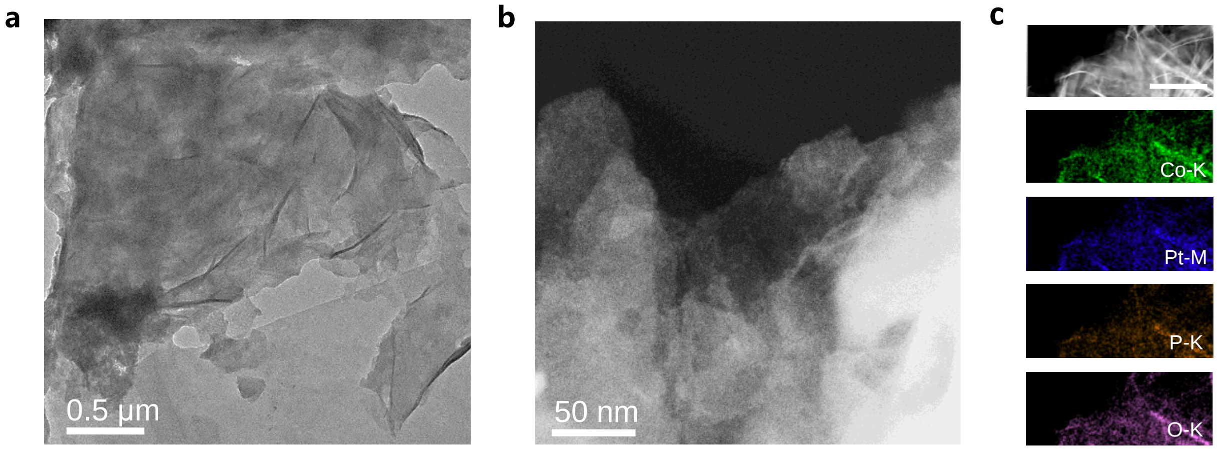

Supplementary Fig. 23 | Structural analysis of Pt_1/CoHPO after OER tests. (a) TEM image, (b) STEM image, (c) elemental mapping of Co, Pt, P and O elements.

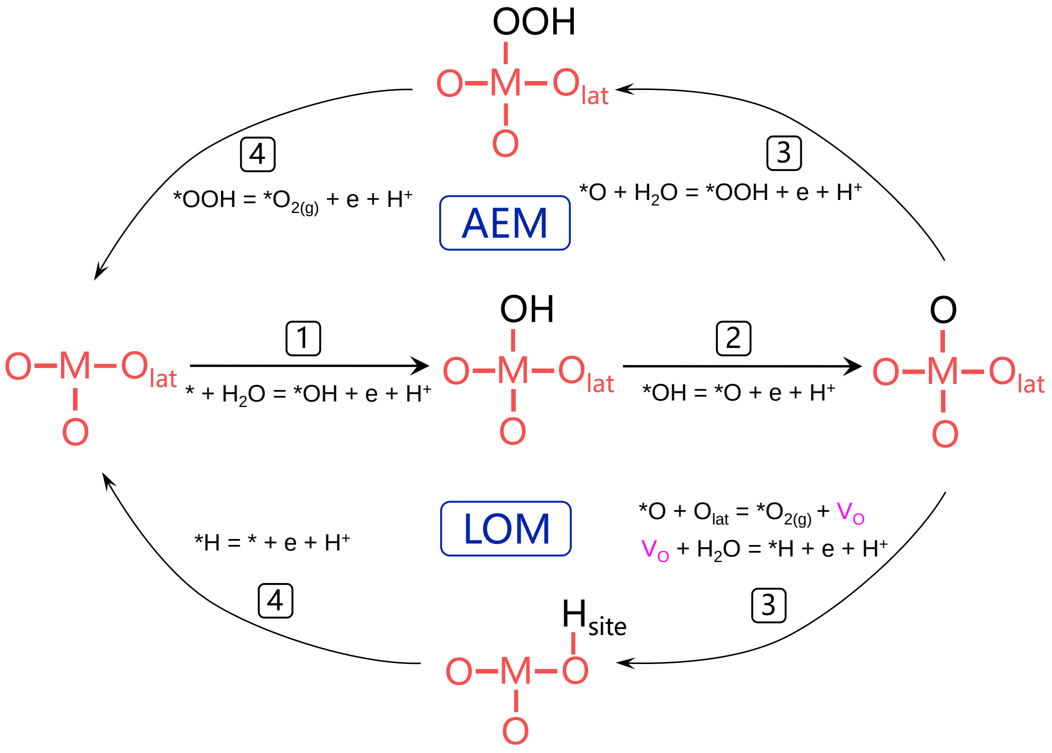

Supplementary Fig. 24 | Schematic of the adsorbate evolving mechanism (AEM) and lattice oxygen mechanism (LOM). * V_O denotes bare surface and lattice oxygen vacancy, respectively. M and O_{lat} stand for metal site and lattice oxygen, respectively.

As for the metal dissolution in electrolyte, it is mainly correlated with the adsorbate evolution mechanisms (AEM) and lattice oxygen activation mechanisms (LOM)[5,6]. Different from the AEM that involves only metal sites and associated H_2O, in the LOM, lattice oxygen can interact with absorbed O_{ad} to form an oxygen vacancy and O_2. However, the existing oxygen vacancies would accelerate the over-oxidation of the active Pt, leading to the highly soluble $Pt^{x>4}$ derivatives (*e.g.*, PtO_3), which is believed to trigger the primary dissolution route. The peak located at ~1083 cm^{-1} can be assigned to the characteristic vibration signal of surface intermediate superoxide species on the Pt surface[7,8]. This suggests the AEM rather than LOM mechanism that dominates O_2 generation over the Pt_1/CoHPO, which improves its structural stability under harsh OER conditions.

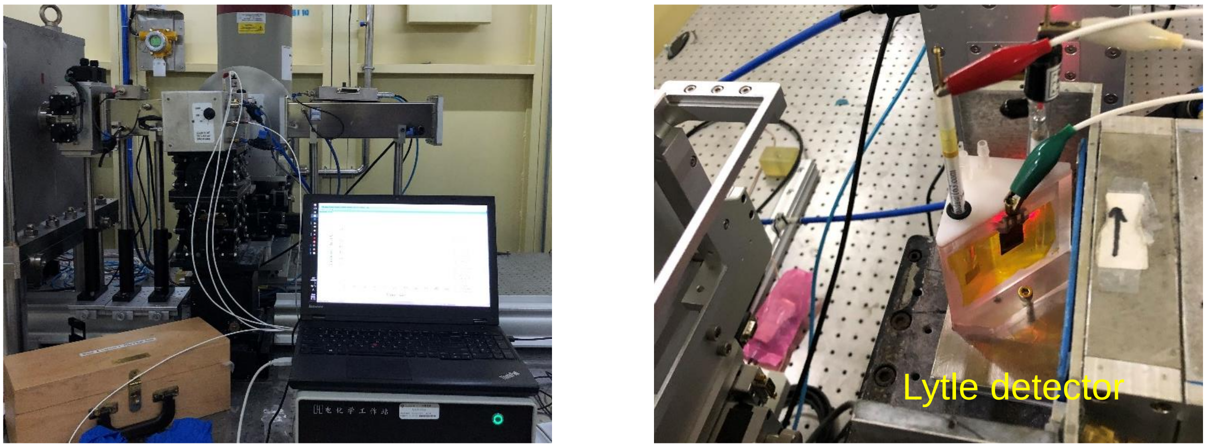

Supplementary Fig. 25 | The device and beam path for XAFS experiments. The experiments were conducted at BL11B in SSRF (Pt L_3-edge and Co K-edge, Lytle detector was used).

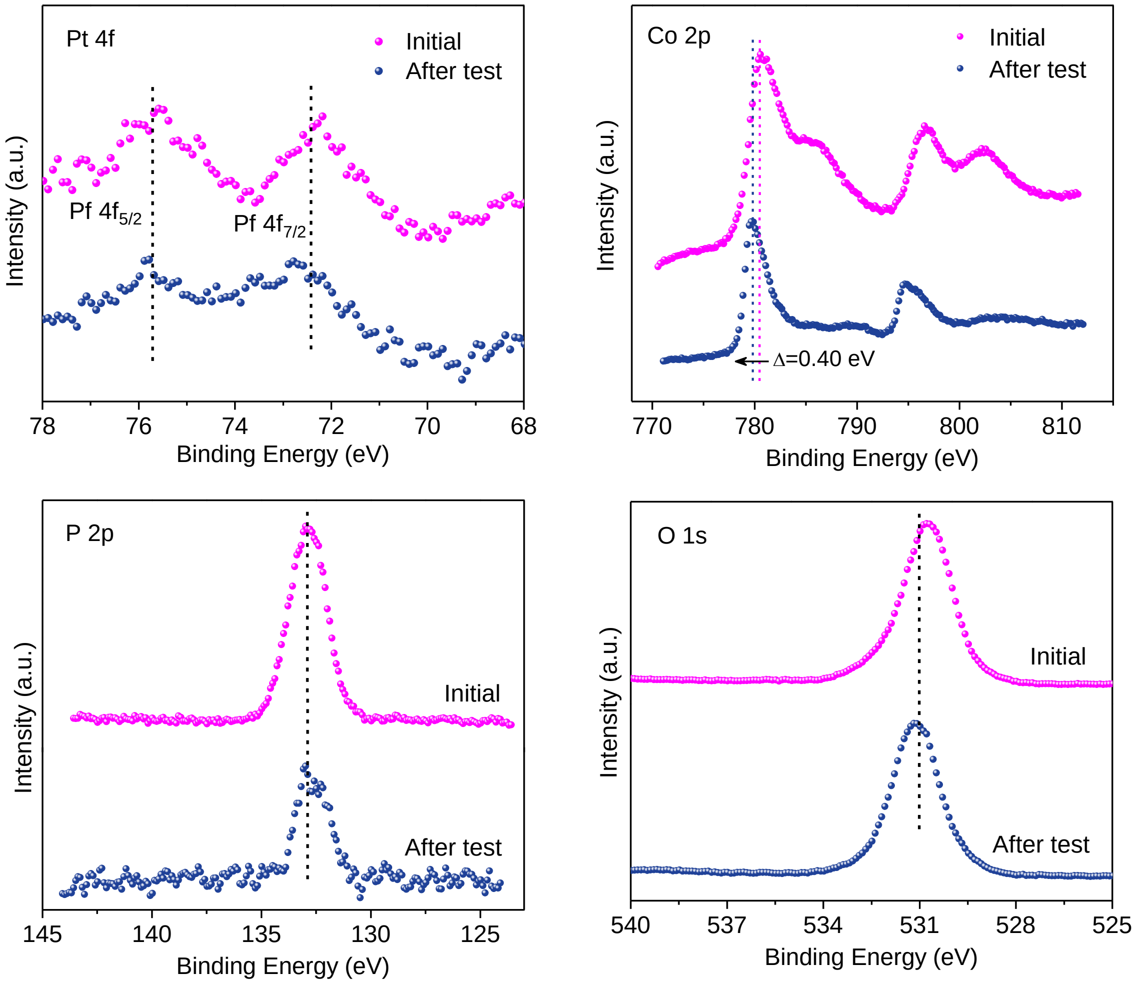

Supplementary Fig. 26 | X-ray photoelectron analyses of Pt_1/CoHPO in 0.1 M KOH after OER. Pt 4f, Co 2p, P 2p and O 1s spectra of Pt_1/CoHPO before and after OER catalysis.

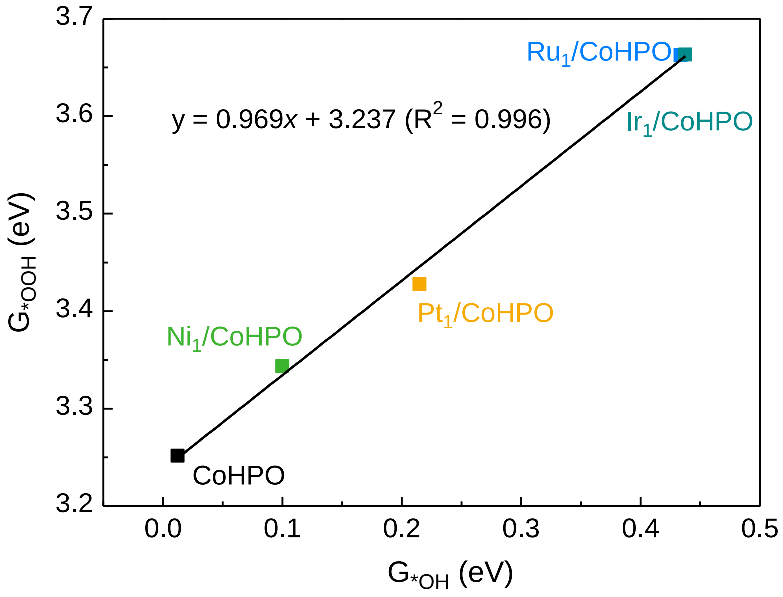

Supplementary Fig. 27 | Additional computational details. The linear scaling relation fitted on the basis of the established M_1/CoHPO models.

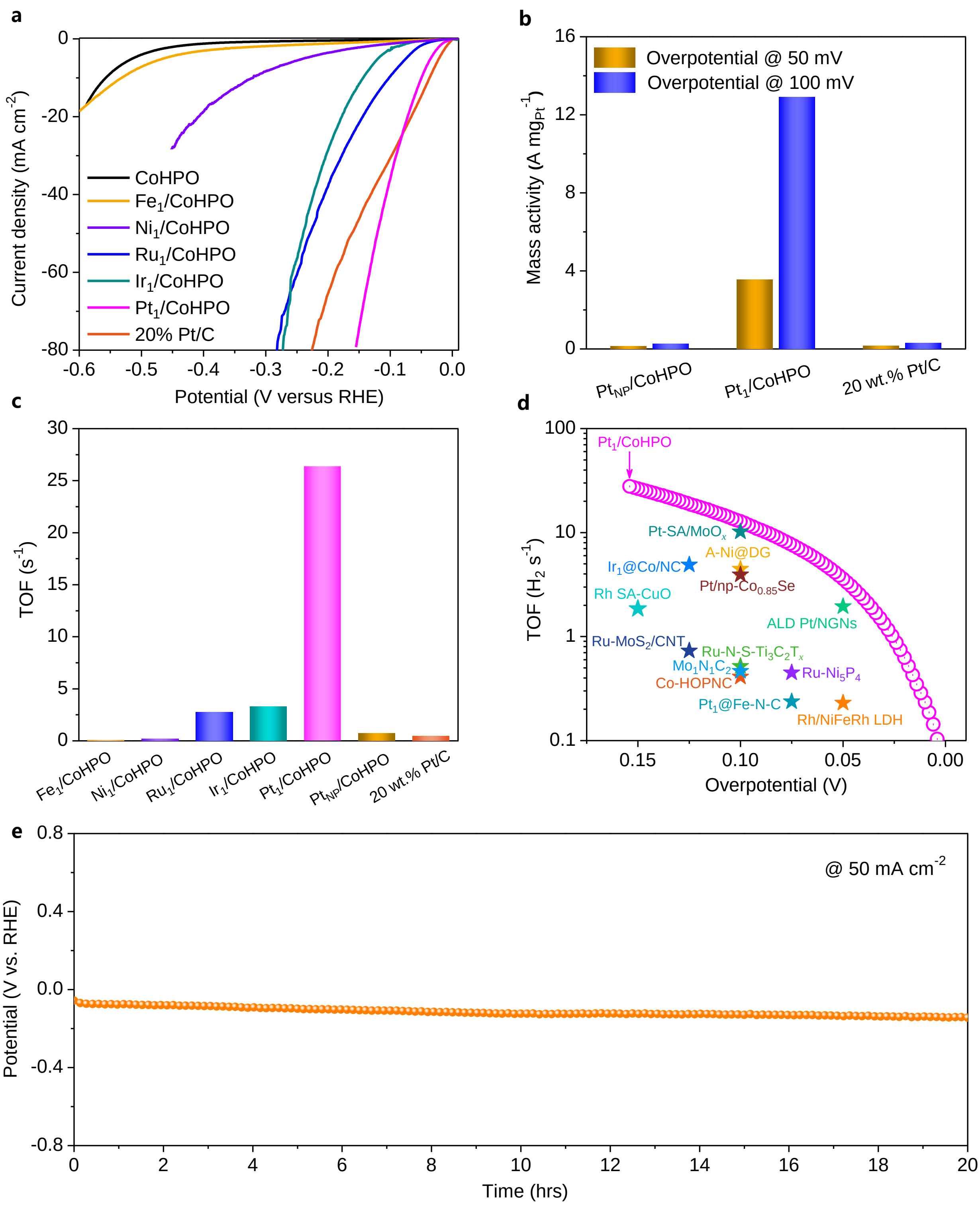

Supplementary Fig. 28 | Catalytic HER performances in 0.1 M KOH. (a) Geometric activity of different catalysts. (b) Mass activity. (c) TOF values at the overpotential of 150 mV. (d) TOF value comparisons of Pt_1/CoHPO with other state-of-the-art electrocatalysts, and the detailed activity comparisons are shown in Supplementary Table 5. (e) Potential-time response at a current density of 50 mA cm^{-2}.

As shown in Supplementary Fig. 28a, the overpotentials delivering 10 mA cm^{-2} current density in 0.1 M KOH follow the sequence: Pt SAs (49 mV) < Ru SAs (100 mV) < Ir SAs (145 mV) < Ni SAs (323 mV) < Fe SAs (525 mV), indicating that the Pt_1/CoHPO achieves the best HER activity. In terms of the utilization of Pt atoms, Pt-based mass activity of Pt_1/CoHPO (12.91 A mg_{Pt}^{-1}) is also far higher than those of Pt_{NP}/CoHPO (0.257 A mg_{Pt}^{-1}) and Pt/C (0.307 A mg_{Pt}^{-1}) catalysts (Supplementary Fig. 28b). Moreover, the calculated TOF value for Pt_1/CoHPO (26.4 s^{-1} per Pt atoms) is 35, 57-fold higher than those of Pt_{NP}/CoHPO (0.74 s^{-1}) and Pt/C (0.46 s^{-1}), respectively, and much higher than other metals (Ru, Ir, Ni, and Fe) single-atoms-loaded CoHPO at the same overpotential of 150 mV (Supplementary Fig. 28c). Impressively, compared with the other state-of-the-art single atom-based HER electrocatalysts, the Pt_1/CoHPO also shows lower overpotential and higher TOF value (Supplementary Fig. 28d and Table 5). Besides, the excellent long-term HER durability of Pt_1/CoHPO was also demonstrated by the negligible increase in potential during a 20 h long-term test (Supplementary Fig. 28e).

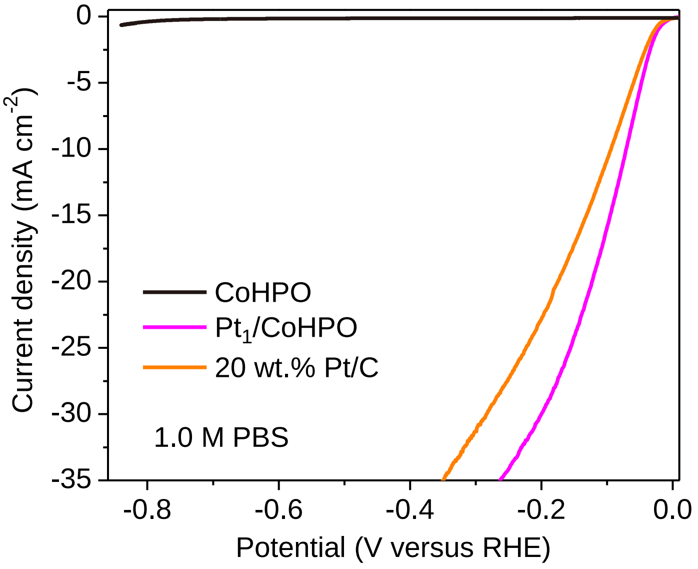

Supplementary Fig. 29 | Catalytic HER performances of different catalysts in neutral media. The polarization curves was conducted in 1.0 M PBS.

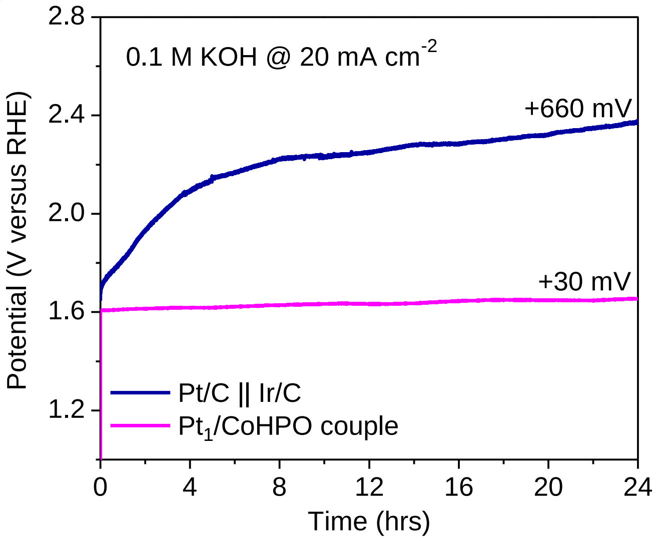

Supplementary Fig. 30 | Overall water splitting stability tests. Potential-time response comparisons of Pt_1/CoHPO couple and benchmark Pt/C || Ir/C as a catalyst in a H-type cell.

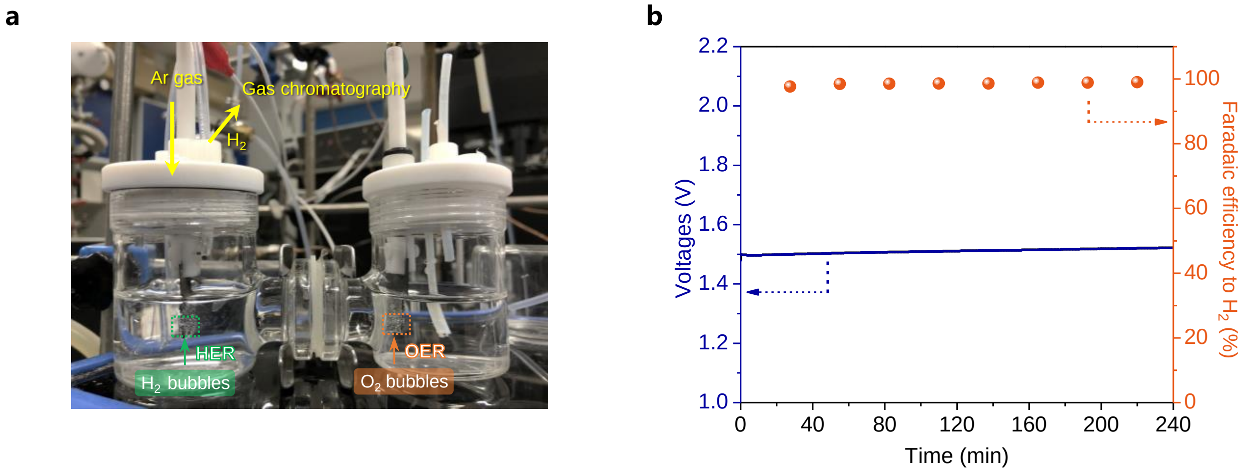

Supplementary Fig. 31 | Hydrogen Faradaic efficiency measurement of the Pt_1/CoHPO. (a) The experiment details of the hydrogen Faradaic efficiency measurement. (b) Faradaic efficiency at 1.5 V toward H_2 and cell voltage-time curve *versus* time in the 240 min measurement.

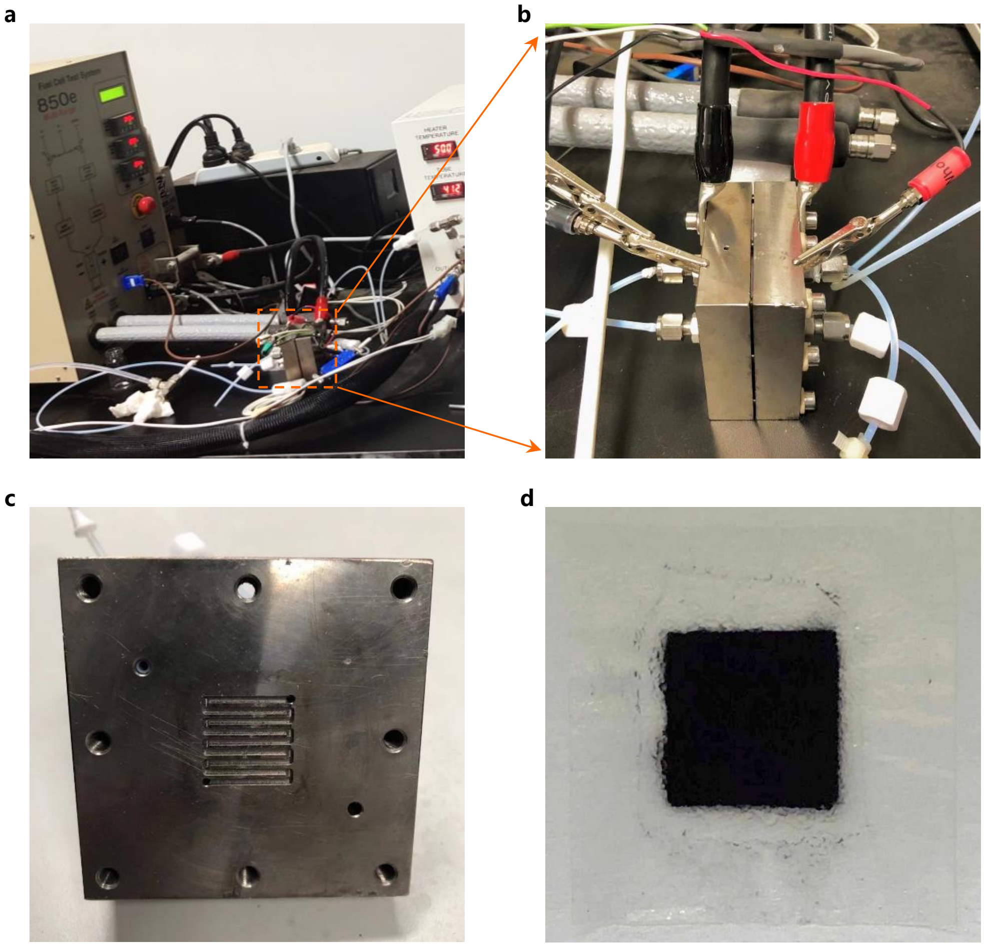

Supplementary Fig. 32 | Photograph of the homemade AEMWEs device. (a, b) Setup of the AEMWEs system. (c) Bipolar plates with a flow field. (d) MEA was prepared by a catalyst-coated membrane (CCM) method.

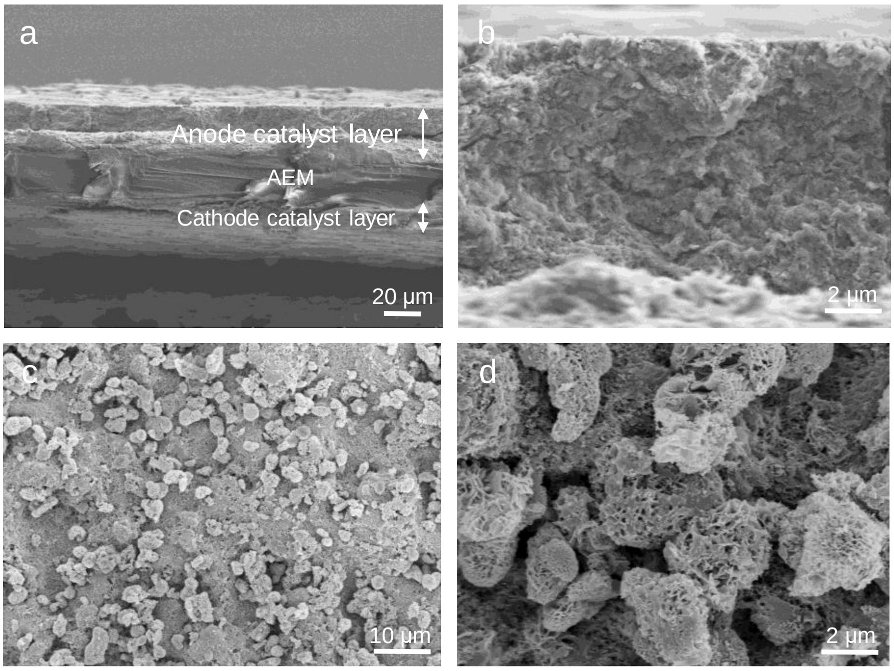

Supplementary Fig. 33 | Electron microscopy of catalyst coated membrane. (a, b) SEM images of cross-section of Pt_1/CoHPO catalysts coated membrane. (c, d) SEM images of top view of Pt_1/CoHPO catalysts coated membrane.

The cross-section SEM image of Pt_1/CoHPO-based MEA is presented in Supplementary Fig. 33a, where the anode, AEM membrane and the cathode are located on the top, middle and bottom of the figure. The respective thicknesses are approximately 30, 40, and 18 μm. It is apparent that the catalyst layers were in intimate contact with the membrane without the formation of any voids (Supplementary Fig. 33b), which would be favorable to the transportation of hydroxide ions. The SEM images of top view (Supplementary Fig. 33c, d) of catalyst layers show a porous surface structure with void spaces between the ultrathin sheet-assembled nanoflower. The large porosity of catalyst layer with more reaction sites would lead to a large roughness factor, which causes effective exchange current density and results in a small activation overpotential[9].

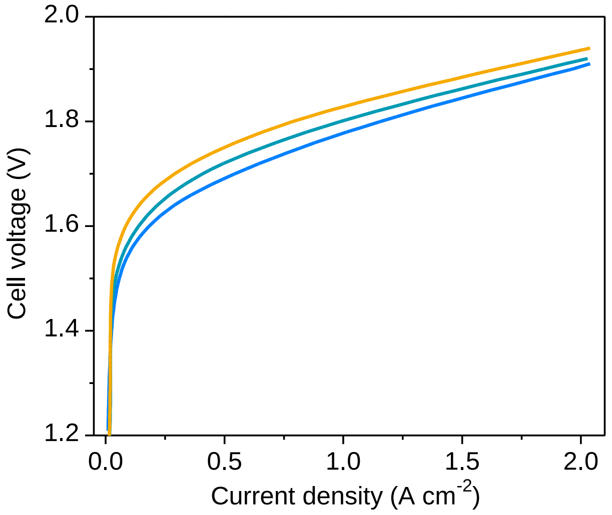

Supplementary Fig. 34 | Independent tests of AEMWE. The current-voltage curves (at 80 ºC) of the Pt_1/CoHPO catalysts-based MEA for three independently integrated and tested cells.

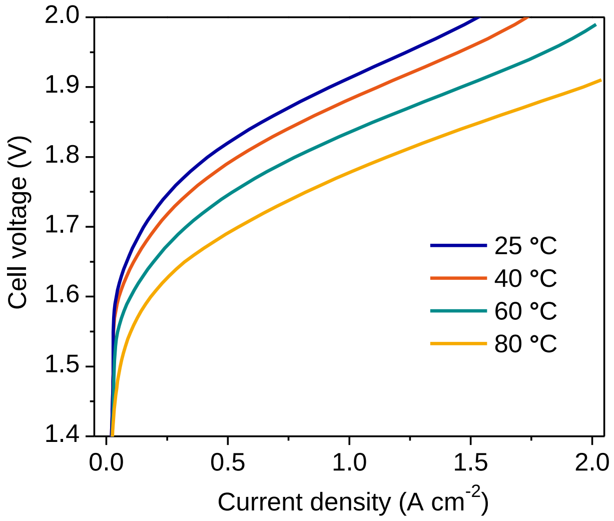

Supplementary Fig. 35 | Effect of operate temperature on the Pt_1/CoHPO-based AEMWEs device performance. As shown in Supplementary Fig. 35, the cell electrolysis voltage decreases with the increasing temperature from 25 to 80 ºC, indicating that the activities of the catalysts are dependent on the operating temperature.

Supplementary Table 1 | Fitting parameters of Pt L_3-edge EXAFS curves[a].

Sample	Path	N	R(Å)	σ^2 (Å^2)	ΔE_0 (eV)	R factor
Pt foil[b]	Pt-Pt	**12**	2.76	0.0044	7.5	0.0024
PtO_2[c]	Pt-O	**6**	2.02	0.0014	13.3	0.0073
Pt_{NP}/CoHPO[d]	Pt-Pt	9.1	2.73	0.0081	5.1	0.008
Pt_1/CoHPO[e]	Pt-O_i	1.0	1.97	0.0026	14.3	0.024
	Pt-O_{ii}	2.6	2.03	0.0026		
	Pt-O-Co	3.7	3.47	0.0033	-4.3	
	Pt-O-P	4.1	2.96	0.0167		

Note: [a]N, coordination number; R, distance between absorber and backscatter atoms; σ^2, Debye-Waller factor to account for both thermal and structural disorders; ΔE_0, inner potential correction; R factor (%) indicates the goodness of the fit. Error bounds (accuracies) that characterize the structural parameters obtained by EXAFS spectroscopy were estimated as $N \pm 20\%$; $R \pm 1\%$;$\sigma^2 \pm 20\%$; $\Delta E_0 \pm 20\%$. S_0^2 was fixed to 0.787 as determined from Pt foil fitting. Bold numbers indicate fixed coordination number (N) according to the crystal structure. [b]Fitting range: $3 \leq k$ (/Å) ≤ 12.46 and $1 \leq R$ (Å) ≤ 3. [c]Fitting range: $3 \leq k$ (/Å) ≤ 12 and $1 \leq R$ (Å) ≤ 2. [d]Fitting range: $3 \leq k$ (/Å) ≤ 12.45 and $1.5 \leq R$ (Å) ≤ 3.2. [e]Fitting range: $3 \leq k$ (/Å) ≤ 10.5 and $1.2 \leq R$ (Å) ≤ 4.0.

Supplementary Table 2 | The calculated Pt-O/OH bond lengths of 5 simulated structural models.

	Pt-O/OH bond length (Å)			
Model-1	1.912	2.036	2.040	2.018
Model-2	2.072	2.000	2.040	2.080
Model-3	2.023	2.035	2.034	1.987
Model-4	2.037	1.855	1.855	
Model-5	2.075	2.092	1.950	1.906

Supplementary Table 3 | Comparison of OER electrocatalytic performance of our Pt_1/CoHPO with recently reported electrocatalysts in recent literatures.

Catalyst	Electrode	Electrolyte	Overpotential at 10 mA cm^{-2} (mV)	Tafel (mV dec^{-1})	TOF at 300 mV (s^{-1})	Mass activity at 300 mV (A mg^{-1})	References
Pt_1/CoHPO	CP[a]	0.1 M KOH	246	49.8	6.81 ± 0.13	13.5 ± 0.3	This work
		1 M KOH	209	41.5	35.2 ± 5.2	69.5 ± 10.3	
A-FeCoW	GCE[b]	1 M KOH	301	N.A.	0.21	N.A.	10
Au/NiFe LDH	Ti mesh	1 M KOH	237	36	NA	0.065 at 280 mV	11
Ir SAs/$NiCo_2O_4$	CP	0.5 M H_2SO_4	240	60	1.13	10 at 237 mV<br>28.89 at 378 mV	12
$Ir_{18wt.\%}$ SAs-NiO	CP	1 M KOH	225	38	1.37	N.A.	13
Ir SA-Ni_2P	GCE	1 M KOH	NA	90.1	NA	11.5	14
$Ir_{0.1}/Ni_9Fe$	Ni foam	1 M KOH	183	49	~9.6	~18.9	15
Co SA-TiO_2	CP	1 M KOH	332	72	6.6	N.A.	16
NiN_4C_4	GCE	1 M KOH	331	64	0.71	N.A.	17
WC_x-FeNi	GCE	1 M KOH	237	44	4.96	14.5	18
$LiCoO_{1.8}Cl_{0.2}$	CP	1 M KOH	270	N.A.	NA	N.A.	19
NiFe-MOF	GCE	0.1 M KOH	240	34	3.8	N.A.	20
IrO_2	CP	1 M KOH	285	75	N.A.	N.A.	21
RuO_2	GCE	1 M KOH	370	105	N.A.	N.A.	22
Ru_1-N_4-C	GCE	0.5 M H_2SO_4	267	52.6	3.72	14.284	23
$Ru_{0.7}WErO_{2-\delta}$	CP	0.5 M H_2SO_4	NA	66.8	NA	1.518	24
$Ru_{2-x}Y_2Co_xO_7$	GCE	1 M KOH	260	34	NA	0.0497	25
Ru_1-Pt_3Cu	GCE	0.1 M $HClO_4$	220	N.A.	NA	6.615 at 280 mV	26
N,S-doped carbon	GCE	1 M KOH	370	273	N.A.	N.A.	27
$CoSe_2$-D_{Fe}-V_{Co}	GCE	1 M NaOH	280	53.3	0.045	N.A.	28
Co_3Ni_1P	GCE	1 M KOH	281	59.6	N.A.	N.A.	29

Note: N.A.: Not available. CP[a]: Carbon paper. GCE[b]: Glassy carbon electrode. All potentials were versus to reversible hydrogen electrode (RHE) with *iR*-correction.

Supplementary Table 4 | Elemental analysis in electrolyte after the chronoamperometry test through ICP measurements.

Sample	Element	Test value/ppm
Electrolyte of 20 wt.% Pt/C (0.5 mg cm^{-2})	Pt	0.2399
Electrolyte of 20 wt.% Pt/C (0.014 mg cm^{-2})	Pt	9.06×10^{-3}
Electrolyte of Pt_1/CoHPO catalyst (0.5 mg cm^{-2})	Pt	$(0.09 \pm 0.05^{\#}) \times 10^{-3}$

#: ± means standard deviation (replicates=3)

To demonstrate Pt_1/CoHPO is more stable than nanoparticle Pt/C under OER operation condition, Pt_1/CoHPO electrode (0.5 mg cm^{-2}) as well as two control electrodes, namely, Pt/C (0.5 mg cm^{-2}) and Pt/C (0.014 mg cm^{-2}) containing the similar Pt mass loading were used. After the chronoamperometry test, we detected the metal dissolution content in the electrolyte *via* ICP-MS measurement. Although Pt/C with a small loading of 0.014 mg cm^{-2} with the identical Pt amount of Pt_1/CoHPO, we note that ca. 9 ppb of Pt was detected in the electrolyte, corresponding to ~60% Pt element used in the electrode. By contrast, Pt content in electrolyte for Pt_1/CoHPO electrode is nearly below the detection limit of ICP-MS (0.1 ppb), suggesting the atomic dispersed Pt in the CoHPO is much more stable than Pt nanoparticle/C during the OER. The great stability of Pt_1/CoHPO arises from the unique Pt_1-O_3/OH_1-Co(P) coordination environment and the amorphous thin nanosheet structure providing abundant defect sites that anchor the atomically dispersed Pt_1 and prevent their dissolution.

Supplementary Table 5 | Comparison of the catalytic HER properties with other state-of-the-art single-atom HER electrocatalysts.

Sample	Single atom	Loading (wt%)	Electrolyte	Overpotential (η) at 10 mA cm^{-2} (mV)	Tafel (mV dec^{-1})	Overpotential (mV) @ TOF (s^{-1})	References
Pt_1/CoHPO	Pt	0.57	0.1 M KOH	49	38.5	50 @ 3.58<br>100 @ 12.8<br>150 @ 26.4	This work
20 wt.% Pt/C		20	0.1 M KOH	37	34.5	50 @ 0.14<br>100 @ 0.31	This work
Pt_1/NMHCS	Pt	1.59	1 M KOH	41	56	100 @ 4.47<br>200 @ 12.0	30
Pt-SA/MoO_x	Pt	0.28	0.5 M H_2SO_4	NA	123	100 @ 10.2	31
Pt/VS_2/CPs	Pt	1.0	0.5 M H_2SO_4	94	40.1	25 @ 0.045#	32
Pt@NHPCP	Pt	3.6	0.1 M $HClO_4$	57	27	25 @ 0.055#	33
Ir_1@Co/NC	Ir	2.2	1 M KOH	55	119	125 @ 4.91#	34
Pt_1@Fe-N-C	Pt	2.1	0.1 M KOH	105	NA	75 @ 0.237#	35
ALDPt/NGNs	Pt	2.1	0.5 M H_2SO_4	50	29	50 @ 1.95	36
Pt/np-$Co_{0.85}$Se	Pt	1.03	1 M PBS	55	35	100 @ 3.93	37
Ru-MoS_2/CNT	Ru	5.0	1 M KOH	50	62	125 @ 0.73#	38
Rh SAC-CuO NAs/CF	Rh	6.8	1 M KOH	44	NA	150 @ 1.85#	39
Rh/NiFeRh-LDH	Rh	4.0	1 M KOH	57	81.3	50 @ 0.23#	40
Ru-Ni_5P_4	Ru	3.83	1 M KOH	54	52	75 @ 0.45#	41
A-Ni@DG	Ni	1.24	1 M KOH	70	45	100 @ 5.7	42
RuSA-N-S-$Ti_3C_2T_x$	Ru	1.2	0.5 M H_2SO_4	76	90	100 @ 0.52	43
Co-SAS-HOPNC	Co	0.49	0.5 M H_2SO_4	137	52	100 @ 0.41	44
$Mo_1N_1C_2$	Mo	1.32	0.1 M KOH	132	90	50 @ 0.148<br>100 @ 0.46	45

Note: NA: Not available, #: The data were calculated according to the polarization curves and the catalyst loading on the electrode given in the literatures. All potentials were versus to reversible hydrogen electrode (RHE) with *iR*-correction.

Supplementary References

1. Liu, Z., *et al.* Selective and controlled synthesis of cobalt hydroxides in highly developed hexagonal platelets. *J. Am. Chem. Soc.* **127**, 13869 (2005).

2. Yu, X., *et al.* Microbiological precipitation, morphology and thermal behavior of barium hydrogen phosphate. *J. Chil. Chem. Soc.* **60**, 2885 (2015).

3. Zhang, Y., *et al.* $Co_3(OH)_2(HPO_4)_2$ as a novel photocatalyst for O_2 evolution under visible-light irradiation. *Catal, Sci. Technol,* **6**, 8080 (2016).

4. Grimaud, A., *et al.* Activating lattice oxygen redox reactions in metal oxides to catalyse oxygen evolution. *Nat. Chem.* **9**, 457 (2017).

5. Wohlfahrt-Mehrens, M.; Heitbaum, J. Oxygen evolution on Ru and RuO_2 electrodes studied using isotope labelling and on-line mass spectrometry. *J. Electroanal. Chem. Interf. Electrochem.* **237**, 251 (1987).

6. Rong, X., *et al.* A fundamental relationship between reaction mechanism and stability in metal oxide catalysts for oxygen evolution. *ACS Catal.* **6**, 1153 (2016).

7. Briega-Martos, V., et al. Detection of Superoxide Anion Oxygen Reduction Reaction Intermediate on Pt (111) by Infrared Reflection Absorption Spectroscopy in Neutral pH Conditions. *J. Phys. Chem. Lett.* **12**, 1588 (2021).

8. Kukunuri, S.; Noguchi, H. In Situ Spectroscopy Study of Oxygen Reduction Reaction Intermediates at the Pt/Acid Interface: Surface-Enhanced Infrared Absorbance Spectroscopy. *J. Phys. Chem. C* **124**, 7267 (2020).

9. Kang, Z., *et al.* Investigation of thin/well-tunable liquid/gas diffusion layers exhibiting superior multifunctional performance in low-temperature electrolytic water splitting. *Energy Environ. Sci.* **10**, 166-175 (2017).

10. Zhang, B., *et al.* Homogeneously dispersed multimetal oxygen-evolving catalysts. *Science* **352**, 333-337 (2016).

11. Zhang, J., *et al.* Single-atom Au/NiFe layered double hydroxide electrocatalyst: probing the origin of activity for oxygen evolution reaction. *J. Am. Chem. Soc.* **140**, 3876-3879 (2018).

12. Yin, J., *et al*. Iridium single atoms coupling with oxygen vacancies boosts oxygen evolution reaction in acid media. *J. Am. Chem. Soc.* **142**, 18378-18386 (2020).

13. Wang, Q., et al. Ultrahigh-loading of Ir single atoms on NiO matrix to dramatically enhance oxygen evolution reaction. *J. Am. Chem. Soc.* **142**, 7425-7433 (2020).

14. Wang, Q., *et al*. Single iridium atom doped Ni_2P catalyst for optimal oxygen evolution. *J. Am. Chem. Soc.* **143**, 13605-13615 (2021).

15. Zheng, X., *et al*. Origin of enhanced water oxidation activity in an iridium single atom anchored on NiFe oxyhydroxide catalyst. *Proc. Natl. Acad. Sci*. **118**, e210817118 (2021).

16. Liu, C., *et al.* Oxygen evolution reaction over catalytic single-site Co in a well-defined brookite TiO_2 nanorod surface. *Nat. Catal.* **4**, 36-45 (2021).

17. Fei, H., *et al*. General synthesis and definitive structural identification of MN_4C_4 single-atom catalysts with tunable electrocatalytic activities. *Nat. Catal.* **1**, 63-72 (2018).

18. Li, S., *et al.* Oxygen-evolving catalytic atoms on metal carbides. *Nat. Mater.* **20**, 1240-1247 (2021).

19. Wang, J., et al. Redirecting dynamic surface restructuring of a layered transition metal oxide catalyst for superior water oxidation. *Nat. Catal.* **4**, 212-222 (2021).
20. Duan, J. et al. Ultrathin metal-organic framework array for efficient electrocatalytic water splitting. *Nat. Commun.* **8**, 1-7(2017).
21. Yan, X., et al. From water oxidation to reduction: transformation from $Ni_xCo_{3-x}O_4$ nanowires to NiCo/NiCoO*x* heterostructures. *ACS Appl. Mater. Interf.* **8**, 3208-3214 (2016).
22. Fu, S., *et al*. Ultrafine and highly disordered Ni_2Fe_1 nanofoams enabled highly efficient oxygen evolution reaction in alkaline electrolyte. *Nano Energy* **44**, 319-326 (2018).
23. Cao, L., *et al*. Dynamic oxygen adsorption on single-atomic Ruthenium catalyst with high performance for acidic oxygen evolution reaction. *Nat. Commun.* **10**, 4849 (2019).
24. Hao, S., *et al*. Dopants fixation of Ruthenium for boosting acidic oxygen evolution stability and activity. *Nat. Commun.* **11**, 5368 (2020).
25. Kim, M., *et al*. Reducing the barrier energy of self-reconstruction for anchored cobalt nanoparticles as highly active oxygen evolution electrocatalyst. *Adv. Mater.* **31**, 1901977 (2019).
26. Yao, Y., *et al.* Engineering the electronic structure of single atomic Ru sites via compressive strain boosts acidic water oxidation electrocatalysis. *Nat Catal.* **2**, 304 (2019).
27. Pei, Z., *et al.* Texturing in situ: N, S-enriched hierarchically porous carbon as a highly active reversible oxygen electrocatalyst. *Energy Environ. Sci.* **10**, 742-749 (2017).
28. Dou, Y., *et al.*, Approaching the activity limit of $CoSe_2$ for oxygen evolution *via* Fe doping and Co vacancy. Nat. Commun. **11**, 1664 (2020).
29. Fu, S. *et al.* Highly ordered mesoporous bimetallic phosphides as efficient oxygen evolution electrocatalysts. *ACS Energy Lett.* 1, 792-796 (2016).
30. Kuang P., *et al.* Pt single atoms supported on N-doped mesoporous hollow carbon spheres with enhanced electrocatalytic H_2-evolution activity. *Adv. Mater.*, **33**, 2008599 (2021).
31. Xu J., *et al.* Amorphous MoO_x-stabilized single platinum atoms with ultrahigh mass activity for acidic hydrogen evolution. *Nano Energy*, **70**, 104529 (2020).
32. Zhu, J., *et al.* Enhanced electrocatalytic hydrogen evolution activity in single-atom Pt-decorated VS_2 nanosheets. *ACS nano* **14**, 5600-5608 (2020).
33. Ying, J., *et al.* Nitrogen-doped hollow porous carbon polyhedrons embedded with highly dispersed Pt nanoparticles as a highly efficient and stable hydrogen evolution electrocatalyst. *Nano Energy* **40**, 88-94 (2017).
34. Lai, W., *et al.* General π-electron-assisted strategy for Ir, Pt, Ru, Pd, Fe, Ni single-atom electrocatalysts with bifunctional active sites for highly efficient water splitting. *Angew. Chem. Int. Ed.* **58**, 11868-11873 (2019).
35. Xiao Z., *et al.*, Single-atom to single-atom grafting of Pt_1 onto $Fe\text{-}N_4$ center: Pt_1@Fe-N-C multifunctional electrocatalyst with significantly enhanced properties. *Adv. Energy Mater.* **8**, 1701345 (2018).
36. Cheng N., *et al.*, Platinum single-atom and cluster catalysis of the hydrogen evolution reaction. *Nat. Commun.* **7**, 13638 (2016).
37. Jiang K., *et al.*, Single platinum atoms embedded in nanoporous cobalt selenide as electrocatalyst for accelerating hydrogen evolution reaction. *Nat. Commun.* **10**, 1743 (2019).

38. Zhang, X., *et al.* Engineering MoS_2 basal planes for hydrogen evolution via synergistic ruthenium doping and nanocarbon hybridization. *Adv. Sci.* **6**, 1900090 (2019).

39. Xu, H., *et al.* Cation exchange strategy to single-atom noble-metal doped CuO nanowire arrays with ultralow overpotential for H2O splitting. *Nano Lett.* **20**, 5482-5489 (2020).

40. Zhang B., *et al*. Integrating Rh species with NiFe-layered double hydroxide for overall water splitting. *Nano Lett.*, **20**, 136 (2020).

41. He Q., *et al.* Achieving efficient alkaline hydrogen evolution reaction over a Ni_5P_4 catalyst incorporating single-atomic Ru sites. *Adv. Mater*., **1**, 1906972 (2020).

42. Zhang L., *et al.* Graphene defects trap atomic Ni species for hydrogen and oxygen evolution reactions. *Chem*, **4**, 285 (2018).

43. Ramalingam V., *et al.* Heteroatom-mediated interactions between ruthenium single atoms and an MXene support for efficient hydrogen evolution. *Adv. Mater.*, **31**, 1903841 (2019).

44. Sun T., *et al.* Single-atomic cobalt sites embedded in hierarchically ordered porous nitrogen-doped carbon as a superior bifunctional electrocatalyst. *Proc. Natl. Acad. Sci.*, **115**, 12692 (2018).

45. Chen W., *et al.* Rational design of single molybdenum atoms anchored on N-doped carbon for effective hydrogen evolution reaction. *Angew. Chem. Int. Ed.*, **56**, 16086 (2017).
